# Arming of MAIT Cell Cytolytic Antimicrobial Activity Is Induced by IL-7 and Defective in HIV-1 Infection

**DOI:** 10.1371/journal.ppat.1005072

**Published:** 2015-08-21

**Authors:** Edwin Leeansyah, Jenny Svärd, Joana Dias, Marcus Buggert, Jessica Nyström, Máire F. Quigley, Markus Moll, Anders Sönnerborg, Piotr Nowak, Johan K. Sandberg

**Affiliations:** 1 Center for Infectious Medicine, Department of Medicine, Karolinska Institutet, Karolinska University Hospital Huddinge, Stockholm, Sweden; 2 Unit of Infectious Diseases, Department of Medicine, Karolinska Institutet, Karolinska University Hospital Huddinge, Stockholm, Sweden; 3 Division of Clinical Microbiology, Department of Laboratory Medicine, Karolinska Institutet, Karolinska University Hospital Huddinge, Stockholm, Sweden; Emory University, UNITED STATES

## Abstract

Mucosa-associated invariant T (MAIT) cells represent a large innate-like evolutionarily conserved antimicrobial T-cell subset in humans. MAIT cells recognize microbial riboflavin metabolites from a range of microbes presented by MR1 molecules. MAIT cells are impaired in several chronic diseases including HIV-1 infection, where they show signs of exhaustion and decline numerically. Here, we examined the broader effector functions of MAIT cells in this context and strategies to rescue their functions. Residual MAIT cells from HIV-infected patients displayed aberrant baseline levels of cytolytic proteins, and failed to mobilize cytolytic molecules in response to bacterial antigen. In particular, the induction of granzyme B (GrzB) expression was profoundly defective. The functionally impaired MAIT cell population exhibited abnormal T-bet and Eomes expression patterns that correlated with the deficiency in cytotoxic capacity and cytokine production. Effective antiretroviral therapy (ART) did not fully restore these aberrations. Interestingly, IL-7 was capable of arming resting MAIT cells from healthy donors into cytotoxic GrzB+ effector T cells capable of killing bacteria-infected cells and producing high levels of pro-inflammatory cytokines in an MR1-dependent fashion. Furthermore, IL-7 treatment enhanced the sensitivity of MAIT cells to detect low levels of bacteria. In HIV-infected patients, plasma IL-7 levels were positively correlated with MAIT cell numbers and function, and IL-7 treatment *in vitro* significantly restored MAIT cell effector functions even in the absence of ART. These results indicate that the cytolytic capacity in MAIT cells is severely defective in HIV-1 infected patients, and that the broad-based functional defect in these cells is associated with deficiency in critical transcription factors. Furthermore, IL-7 induces the arming of effector functions and enhances the sensitivity of MAIT cells, and may be considered in immunotherapeutic approaches to restore MAIT cells.

## Author Summary

The mucosa-associated invariant T (MAIT) cells recognize antigens that are byproducts of the riboflavin biosynthesis pathway shared by many microbes. These antigens are presented by the MHC class I-like MR1 molecules and trigger rapid activation of MAIT cells in an innate-like fashion with deployment of effector mechanisms including cytokine production and cytolysis. Here, we investigated the MAIT cell response to bacteria in humans infected with HIV-1, and possible means to restore functionality to these cells. MAIT cell dysfunction in HIV-infected patients included an inability to express components of the cytolytic effector machinery. Impairment of the MAIT cell population involved the loss of expression of the transcription factors T-bet and Eomes. Interestingly, IL-7 had strong effects on MAIT cells, including the antigen-independent arming of cytolytic function and enhanced sensitivity for low levels of bacteria. In HIV-infected patients, plasma IL-7 levels were positively associated with the size of the MAIT cell population, and IL-7 could rescue their function. These findings indicate that MAIT cell impairment in HIV-1 infection is broad-based, includes loss of critical transcription factors, and loss of cytolytic function. Furthermore, the data support the notion that IL-7 is a strong candidate for immunotherapy in diseases associated with MAIT cell loss.

## Introduction

Mucosa-associated invariant T (MAIT) cells are a recently described subset of unconventional, innate-like T cells that are highly abundant in mucosal tissues, liver and circulation of healthy humans [1–4]. MAIT cells express a semi-invariant T cell receptor (TCR), including Vα7.2 coupled with restricted Jα segments (Jα33, Jα12, or Jα20), and limited Vβ repertoires [5, 6]. Together with their semi-invariant TCR, human MAIT cells are also defined by their high expression of CD161, the IL-18 receptor α subunit (IL-18Rα), and the transcription factor ZBTB16 [7], also known as promyelocytic leukemia zinc finger protein (PLZF) [8, 9]. The vast majority of MAIT cells are either CD8αα or CD8αβ, with some CD4/8 double-negative (DN), and minor CD4^+^ populations [8–11]. Human MAIT cells acquire innate-like antimicrobial activity in the fetal intestinal mucosa pre-natally, prior to the establishment of the commensal microflora [12].

MAIT cells recognize antigens in complex with the MHC-Ib-related protein (MR1) [2, 4], which displays an extraordinary level of evolutionary conservation among placental and marsupial mammals [4, 13, 14]. MR1 presents microbial vitamin B_2_ (riboflavin) metabolites from a wide range of microbes [15, 16], including unstable intermediates that are formed from non-enzymatic condensation of the early intermediate of riboflavin biosynthesis 5-amino-6-D-ribitylaminouracil (5-A-RU) with host- or microbe-derived glyoxal or methylglyoxal [15, 17]. MR1 captures these otherwise unstable compounds and presents them to MAIT cells [17]. Once activated by antigens, MAIT cells can rapidly kill infected cells [18, 19], inhibit intracellular microbial growth [20], and produce pro-inflammatory cytokines including IFNγ, TNF, and IL-17 [8, 10–12, 21]. Certain innate cytokines, including IL-12 and IL-18, can stimulate MAIT cells to produce IFNγ independently of the MR1-TCR interaction [22, 23]. These findings are strongly supportive of the notion that MAIT cells perform critical functions in the immune system, also beyond their role as antimicrobial T cells, particularly at mucosal sites.

Despite the great advances of antiretroviral therapy (ART) in the management of HIV disease, infected patients are still at an increased risk of microbial co-infections such as *Mycobacterium tuberculosis*, non-typhoidal *Salmonella* and *Streptococcus pneumoniae* [24–27]. Such microbial co-infection burdens are particularly apparent in individuals who are diagnosed at advanced stages, lack access to ART, and those who are non-adherent to therapy and clinical care. Although MAIT cells do not directly recognise viral antigens, indirect involvement of these cells in viral immunopathogenesis can occur, as we originally and others recently described for HIV-1 infection [11, 21, 28–31]. MAIT cell numbers and Th1/17 cytokine production are severely and persistently reduced in chronic HIV-infected patients [11]. The polymicrobial reactivity and breadth of the MAIT cell functional profile most likely contribute to the reported role of MAIT cells in the protection against diverse bacterial and mycobacterial infections in animal models, as well as in severe bacterial infections and pulmonary tuberculosis in humans [8, 20, 32–35]. MAIT cell defects may therefore predispose HIV-infected patients to an increased risk of acquiring microbial co-infections. Furthermore, the loss of MAIT cells is potentially irreversible if the disease is not treated at a very early stage [11, 21, 28–31]. In most settings of HIV-1 infection early diagnosis and treatment is challenging, and this necessitates a strategy where MAIT cells can be rescued through adjunctive immunotherapies.

IL-7 is a pleiotropic cytokine that has strong survival and homeostatic effects towards T cells, particularly the memory T cell populations with which MAIT cells show similarities (reviewed in [36]). IL-7 has attracted interest in the context of HIV-1 disease, and was proposed as a potential cytokine intervention therapy in treatment failure as well as in approaches to purge the viral reservoir (reviewed in [37]). Furthermore, IL-7 was recently shown by Tang et al. to enhance MAIT cell Th1/17 cytokine production in response to polyclonal stimulation [38]. In the present study, we investigate the cytolytic function of MAIT cells in the context of HIV-1 infection, the basis for dysfunction of MAIT cells in this disease, and possible approaches to rescue their function. Our findings indicate that arming of cytolytic capacity in MAIT cells is severely defective in HIV-1 infected patients, and that the broad-based functional defects in these cells are associated with deficiency in critical transcription factors. Furthermore, IL-7 induces the arming of effector functions and enhances the sensitivity of MAIT cells in healthy donors, and can partially reverse the functional and transcription factor defects in MAIT cells from HIV-1 infected patients. Thus, inclusion of IL-7 may be considered in immunotherapeutic approaches to restore MAIT cell numbers and function in conditions associated with loss and dysfunction of these cells.

## Results

### MAIT cells express cytolytic proteins at steady state and become rapidly armed into full effector T cells following bacterial stimulation

**Fig 1:**
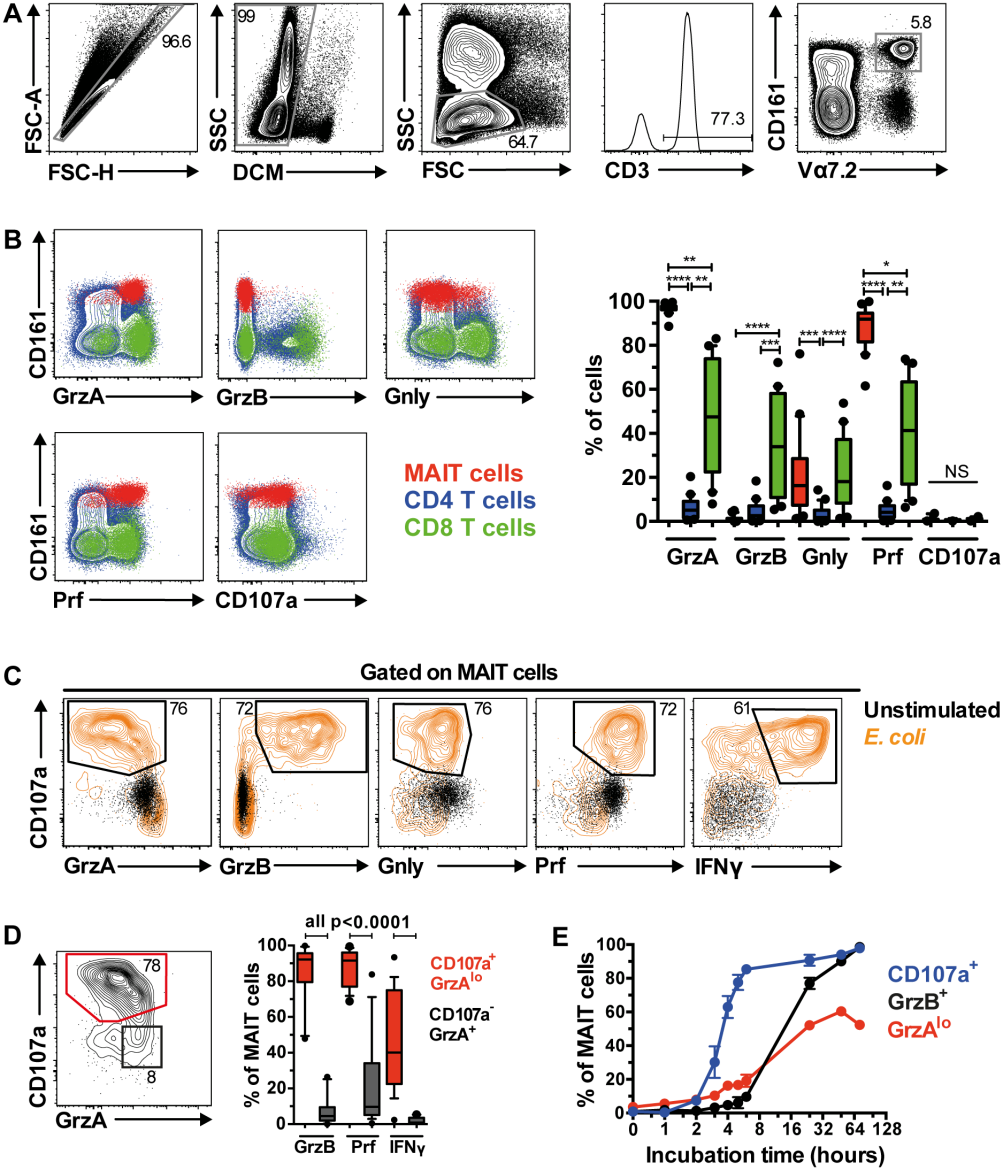
MAIT cells are rapidly armed into full effector T cells following bacterial stimulation. (A) The gating strategy to identify Vα7.2^+^CD161^hi^ MAIT cells through flow cytometry. (B) The comparison of cytolytic proteins (granzyme A (GrzA), granzyme B (GrzB), granulysin (Gnly)), pore-forming protein perforin (Prf), and degranulation marker (CD107a) expression by MAIT cells (red), and CD4^+^ (blue) and CD8^+^ (green) conventional T cells at resting state from 20 HIV-uninfected, healthy controls. (C) Expression of cytolytic proteins by MAIT cells following stimulation with PFA-fixed *E*. *coli* (orange contour plot) or left unstimulated for 24 h (black dot plot). (D) Activated CD107a^+^GrzA^lo^ MAIT cells (red contour plot) following bacterial stimulation expressed high levels of GrzB, Prf, and IFNγ, whereas the non-activated CD107a^-^GrzA^+^ MAIT cells (black dot plot) were variable for Prf expression and mostly negative for GrzB and IFNγ (n = 20). (E) The kinetics of MAIT cell expression for CD107a (blue line), GrzA^lo^ (red line), and GrzB (black line) after PFA-fixed *E*. *coli* stimulation (n = 3). Error bars represent mean and standard deviation. Representative FACS plots from at least 5 healthy donors are shown. Box and whisker plot shows median, IQR and the 10^th^ to the 90^th^ percentile. The Friedman test followed by Dunn’s post-hoc test was used to detect significant differences across multiple, paired samples (B), and the Wilcoxon signed rank test for paired samples (D). NS: not significant (p≥0.05), *p<0.05, **p<0.01, ***p<0.001, ****p<0.0001.

The expression levels of the cytolytic proteins perforin (Prf), granzyme (Grz)A, GrzB, and granulysin (Gnly), as well as the degranulation marker CD107a, were investigated in unstimulated MAIT cells obtained from 20 HIV-uninfected healthy controls (Fig 1A and 1B), and compared with those of non-MAIT, conventional CD4^+^ and CD8^+^ T cells (Fig 1B). The majority of MAIT cells expressed Prf and GrzA, whereas Gnly was expressed only by a proportion of MAIT cells and displayed high inter-donor variability (Fig 1B). Interestingly, MAIT cells did not express GrzB at steady state (Fig 1B). This pattern of cytolytic protein expression by MAIT cells was significantly different from non-MAIT, conventional CD4^+^ and CD8^+^ T cells (Fig 1B), consistent with recent studies [18, 19]. In addition, GrzA and Gnly in MAIT cells were co-expressed with the pore-forming protein Prf (S1A Fig).

We next determined MAIT cell cytolytic protein expression profile following overnight stimulation with mildly PFA-fixed *E*. *coli* in 20 healthy individuals, as we have previously shown that such stimulation triggers robust expression of pro-inflammatory cytokines in MAIT cells [11, 12]. Following stimulation, MAIT cells expressed high levels of the degranulation marker CD107a, coupled with loss of Gnly and GrzA expression, a concomitant upregulation of GrzB and Prf, as well as IFNγ production (Fig 1C). Furthermore, the high Prf and GrzB expression levels were predominantly found within the CD107a^+^GrzA^lo^ MAIT cell population (Fig 1D), indicating that MAIT cells exocytose these cytolytic proteins following bacterial stimulation. Interestingly, not all CD107a^+^GrzA^lo^ MAIT cells produced IFNγ (Fig 1D), suggesting that some functional heterogeneity within the MAIT cell population may exist. MAIT cell degranulation occurred rapidly, with detectable CD107a expression within two hours following bacterial feeding, followed by the appearance of GrzA^lo^ MAIT cells, and then finally GrzB upregulation within 24 h post-stimulation with optimal upregulation at 72 h post-stimulation (Fig 1E). Together, these results indicate that MAIT cells rapidly mobilize their cytolytic granules upon antigen stimulation, and upregulate GrzB expression to become fully armed effector T cells.

### MAIT cells express a distinct combination of transcription factors

**Fig 2:**
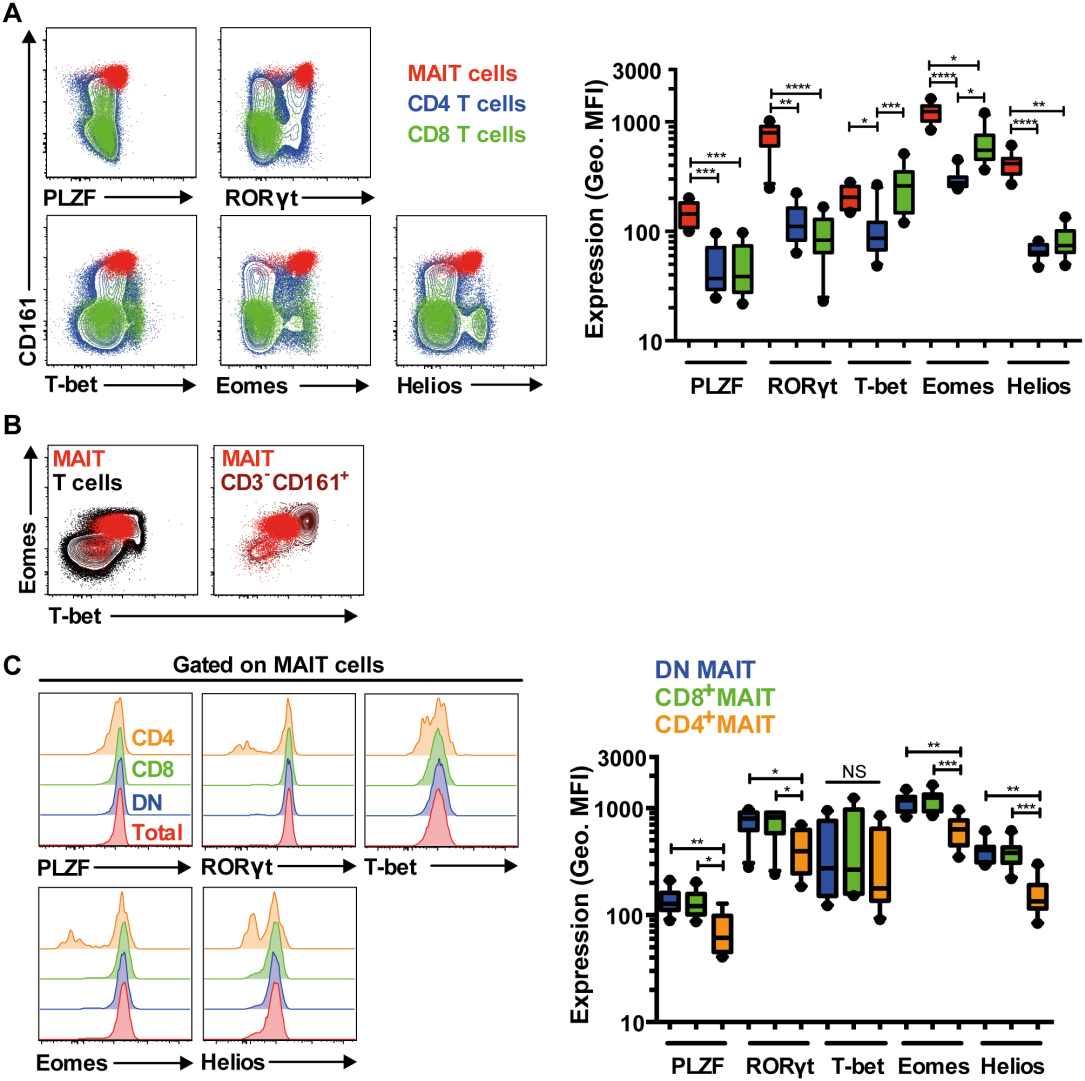
MAIT cells express a distinct transcription factor profile. (A) Freshly isolated PBMCs from 10 HIV-uninfected, healthy controls were stained for the transcription factors PLZF, RORγt, T-bet, Eomes, and Helios. The expression levels for these transcription factors were determined and compared in MAIT cells (red), and in conventional CD4^+^ (blue) and CD8^+^ (green) T cells. (B) MAIT cells (red) T-bet:Eomes expression ratio when compared to conventional T cells (black) and CD3^-^CD161^+^ lymphocytes (brown). MAIT cells express a low T-bet: high Eomes ratio (T-bet^dim^Eomes^hi^). (C) The transcription factor expression levels among the CD4^-^CD8^-^ DN (blue) and CD8^+^ MAIT cell populations (green) that comprised the vast majority of total MAIT cells (typically >95%; red) were similar. Variable expression levels within the small minority CD4^+^ MAIT cells (orange) were observed. Representative FACS plots are shown. Box and whisker plots show median, IQR and the 10^th^ to the 90^th^ percentile. *p<0.05, **p<0.01, ***p<0.001, ****p<0.0001 (the Friedman test followed by Dunn’s post-hoc test).

Recent studies show that MAIT cells express the innate-like T cell transcription factor PLZF and the Th17 master transcription factor RORγt, which are likely to be responsible for the effector memory-like and Th17-like phenotype in MAIT cells, respectively [3, 8, 9]. However, the MAIT cell expression profile of other T cell transcription factors is unknown, including T box transcription factor 21 (TBX21, or T-bet), Eomesodermin (Eomes), and Helios. Therefore, the intracellular expression of PLZF, RORγt, T-bet, Eomes, and Helios was investigated in peripheral blood MAIT cells from 10 healthy donors. As expected, MAIT cells expressed PLZF and RORγt (Fig 2A). PLZF and RORγt co-expression in CD8^+^ T cells, total T cells, and to a lesser extent, in DN T cell populations accurately identified the MAIT cell population (S1B Fig), whereas far fewer of either PLZF^+^RORγt^-^ or PLZF^-^RORγt^+^ cells from any T cell population were MAIT cells (S1B Fig). In addition, MAIT cells expressed the classical effector T cell transcription factors T-bet and Eomes, as well as Helios, with low expression levels for T-bet, high levels for Eomes, and intermediate levels for Helios (Fig 2A). MAIT cells also appeared to express lower levels of T-bet coupled with higher levels of Eomes (T-bet^dim^ Eomes^hi^) when compared to conventional T cells and CD3^-^ CD161^+^ lymphocytes (predominantly NK cells) (Fig 2B). The MAIT cell expression level patterns for transcription factors were distinct to those expressed by conventional CD4^+^ and CD8^+^ T cells (Fig 2A), and were homogeneous across the predominant DN and CD8^+^ MAIT cell subsets, although lower expression levels were observed in the minor CD4^+^ MAIT cell subset (Fig 2C).

### IL-7 arms MAIT cells and potently enhances MAIT cell cytolysis of target cells

**Fig 3:**
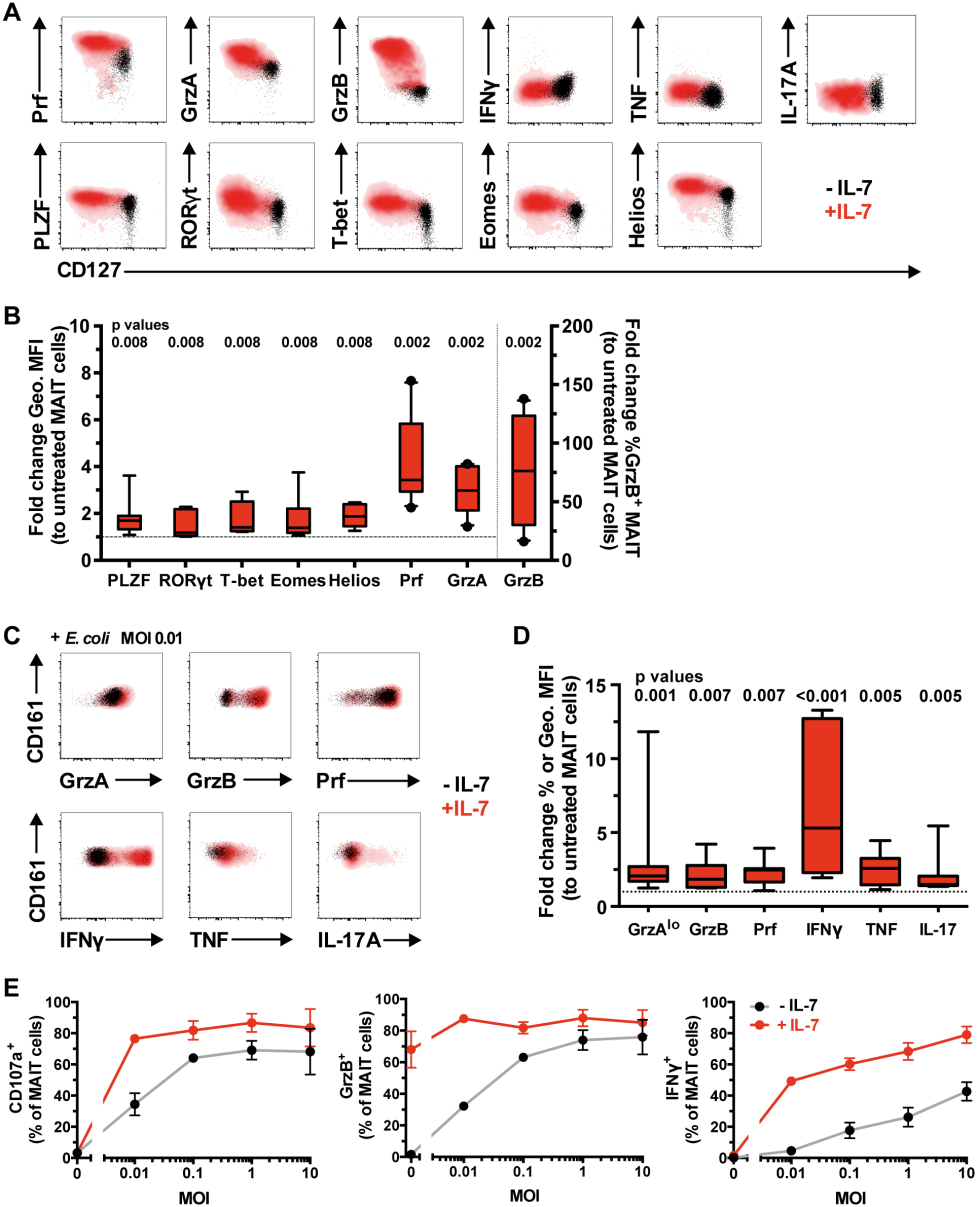
IL-7 arms MAIT cell cytotoxic capacity. (A) Freshly isolated PBMCs from healthy blood donors were left untreated (black) or treated with IL-7 (red) for 48 h, and the expression levels of cytolytic proteins, pro-inflammatory cytokines, and innate and classical T cell transcription factors were determined in MAIT cells without bacterial stimulation (A), where the levels of cytolytic proteins (n = 10) and transcription factors (n = 8) expressed by IL-7-treated MAIT cells were then compared to those of untreated cells (B), or (C) after stimulation with a low dose of PFA-fixed *E*. *coli* (n = 8). (D) Fold-change geometric MFI was measured for Prf levels, and fold-change frequency for the rest of cytolytic proteins and cytokines. (E) Dose-response curve for MAIT cell degranulation (CD107a), cytotoxic capacity (GrzB), and pro-inflammatory cytokine production (IFNγ) after PFA-fixed *E*. *coli* stimulation in untreated (black line) or IL-7-treated (red line) MAIT cells (n = 3). Representative FACS plots are shown. Box and whisker plots show median, IQR and the 10^th^ to the 90^th^ percentile. Dotted line indicates the normalised levels of transcription factors or cytolytic proteins expressed by IL-7-untreated MAIT cells. The Wilcoxon signed rank test (B) or paired t-test (D) was used to determine significance between IL-7-untreated and-treated samples. Error bars represent mean and standard deviation.

A recent study showed that IL-7 can enhance MAIT cell production of Th1/17 cytokines following CD3/CD28 stimulation [38]. Here, we assessed whether IL-7 could enhance MAIT cell antimicrobial cytotoxic potential in healthy individuals. Following a 48 h stimulation with IL-7 alone, the expression levels of Prf and GrzA were elevated and GrzB expression was induced, and this occurred in the absence of concurrent production of Th1/17 cytokines (Fig 3A and 3B). Such induction of cytolytic effector molecules by MAIT cells reached their maxima when incubated with 5–25 ng/ml of IL-7 (S2A Fig). There was no significant effect of IL-7 on Gnly expression in resting MAIT cells (S2B Fig). The induction of cytolytic effector molecule expression in MAIT cells by IL-7 was MR1-independent as determined by MR1 blocking using the 26.5 mAb (S2B Fig). Interestingly, IL-7 enhanced the expression of PLZF, RORγt, T-bet, Eomes, and Helios in MAIT cells examined in eight healthy individuals (Fig 3A and 3B). In contrast, there was no upregulation of Ki67 expression following IL-7 treatment, indicating that IL-7 did not trigger proliferation of MAIT cells (S2C Fig).

We next examined the effect of IL-7 treatment on MAIT cell effector responses following overnight stimulation with a suboptimal dose of mildly fixed *E*. *coli* (MOI 0.01). IL-7 treatment significantly enhanced expression of MAIT cell cytotoxic effector molecules and pro-inflammatory cytokine production as compared to MAIT cells exposed to bacteria alone (Fig 3C and 3D). In addition, IL-7 boosted the sensitivity of MAIT cells and allowed degranulation, GrzB upregulation, and IFNγ production at an antigenic dose up to 1000-fold lower when compared to IL-7-untreated MAIT cells (Fig 3E). The enhancement of MAIT cell effector function in response to *E*. *coli* stimulation reached its maximum when treated with 5–25 ng/ml IL-7 (S2D Fig).

IL-18 and IL-12 have been shown to stimulate MAIT cell effector function [19, 22, 23]. We therefore compared these cytokines with IL-7 in their capacity to arm MAIT cell effector function. In three HIV-uninfected donors, IL-7 was similar or superior to IL-18/IL-12 in arming and enhancing MAIT cell effector functions alone or following bacterial stimulation (S3A and S3B Fig). The exception to this pattern was IFNγ, as IL-12/IL-18 triggered direct activation of this effector function both alone and together with *E*. *coli* stimulus (S3A and S3B Fig). Furthermore and contrary to IL-7 treatment, IL-18/IL-12 seemed to enhance the decrease in MAIT cell numbers following bacterial stimulation (S3C Fig). This observation is in line with previous studies where IL-18 and IL-12 triggered direct effector responses and were implicated in MAIT cell loss [30, 39].

**Fig 4:**
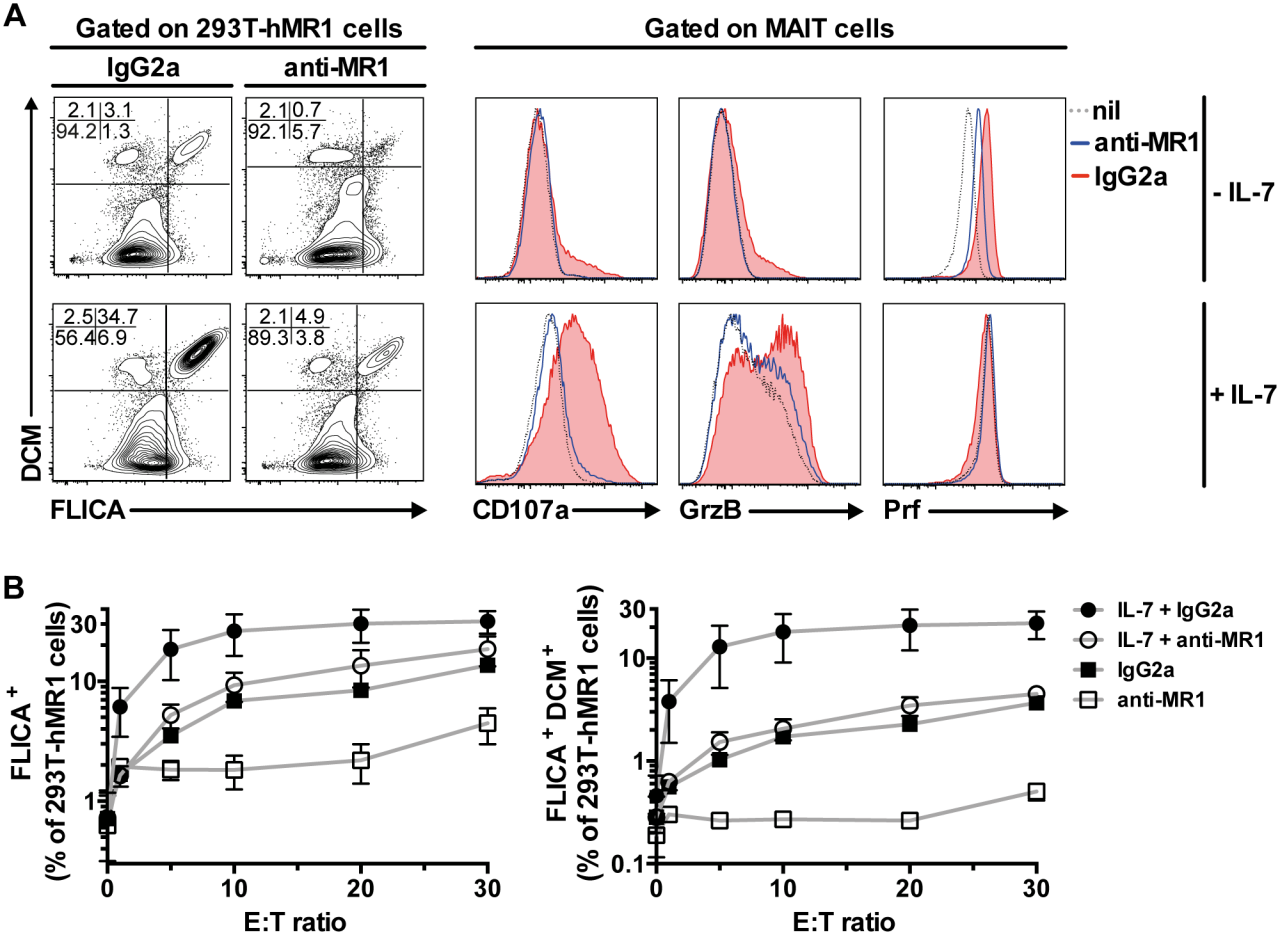
IL-7 potently enhances MAIT cell killing of bacteria-exposed cells. (A) The effect of IL-7 on MAIT cell killing of bacteria-exposed cells was determined using 293T-hMR1 cells that were pulsed with PFA-fixed *E*. *coli* (MOI 10) and co-cultured with purified MAIT cells that were previously untreated or treated with IL-7 for 72 h. Anti-MR1 or IgG2a isotype controls were added to determine MR1-dependency of MAIT cell killing of bacteria-exposed cells. 293T-hMR1 cell death was defined as cells that were positive for both poly-caspases activities (FLICA^+^) and amine-reactive live/dead cell marker (DCM^+^). MAIT cell degranulation, and expression of GrzB and Prf were simultaneously assessed. (B) The effector to target cells (E:T) ratio curve for MAIT cell killing of PFA-fixed *E*. *coli*-pulsed 293T-hMR1 cells was determined in 3 independent donors. FLICA^+^ cells denote total apoptotic cells (left panel), whereas FLICA^+^DCM^+^ cells denote dead cells (right panel). Representative FACS plots are shown. Error bars represent mean and standard deviation.

Because IL-7 treatment armed MAIT cells to become GrzB-expressing effector T cells, we investigated the effect of IL-7 on MAIT cell-mediated killing of bacteria-fed cells. Here, we utilized an *in vitro* model system where human MR1-expressing 293T (293T-hMR1) cells were fed mildly fixed *E*. *coli* and co-cultured with MAIT cells. Untreated MAIT cells were not able to significantly kill bacteria-fed target cells, whereas IL-7 treatment boosted MAIT cell killing of target cells by about 10-fold (Fig 4A and 4B). In resting IL-7 untreated cells, there was a correlation between degranulation and GrzB co-expression (CD107a^+^ GrzB^+^) by effector MAIT cells and target cell killing following exposure to *E*. *coli* (S3D Fig). This pattern indicated that the lackluster killing of target cells by IL-7 untreated MAIT cells was due to the low levels of degranulation and GrzB (Fig 4). In contrast, MAIT cells that were treated with IL-7 expressed GrzB, readily degranulated and had even higher GrzB levels upon co-culture with bacteria-fed target cells (Fig 4A). Both IL-7-treated and-untreated MAIT cell killing of target cells was ablated by anti-MR1, suggesting that MAIT cell killing is predominantly MR1-dependent (Fig 4A and 4B). Taken together, these results indicate that IL-7 arms MAIT cells to become effector T cells and potently induces MR1-dependent killing capacity through the upregulation of GrzB expression as well as other effector proteins, and this occurs concomitantly with elevated expression of transcription factors including T-bet and Eomes.

### MAIT cell arming and cytotoxic capacity is impaired in chronic HIV-1 infection

**Fig 5:**
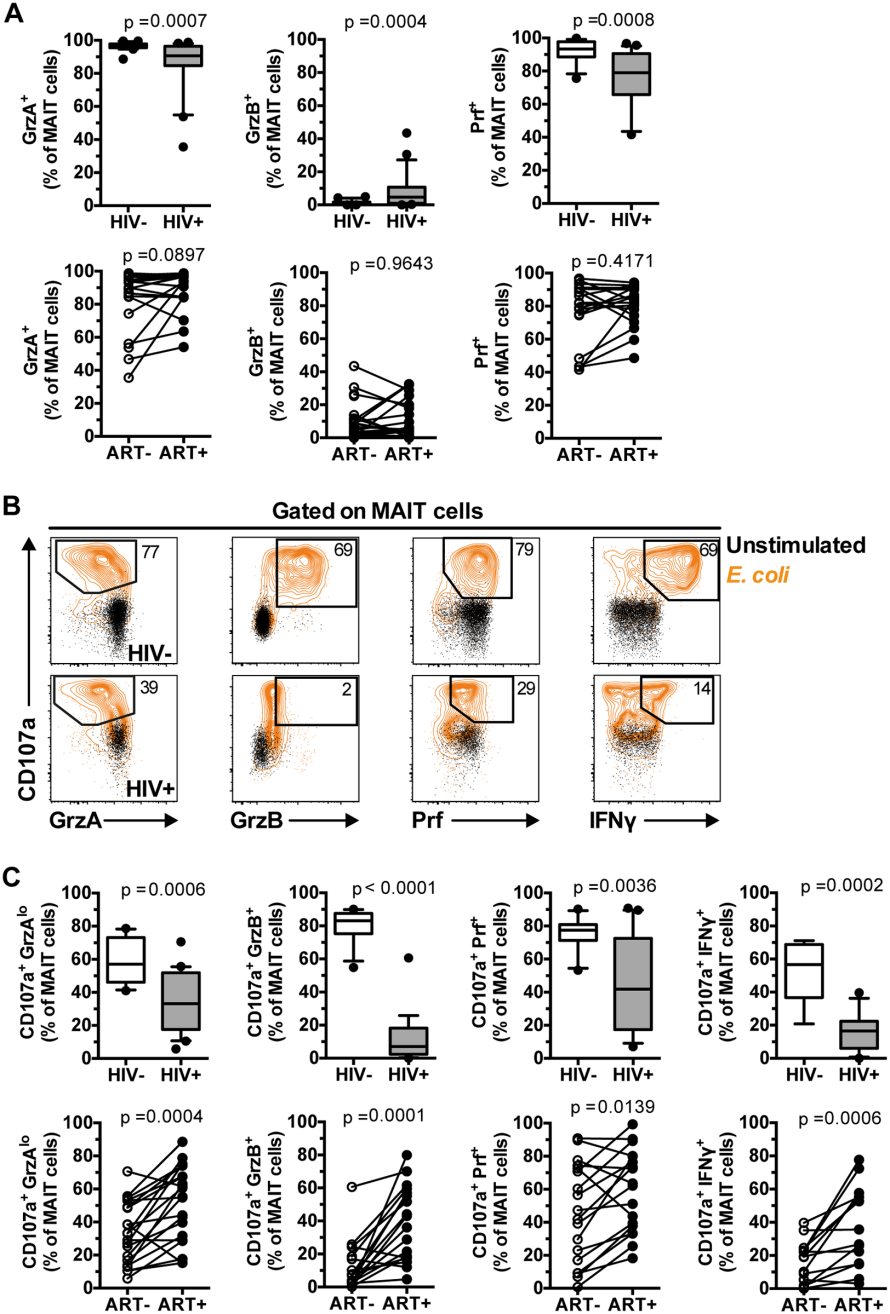
IL-7 arming of MAIT cells and MAIT cell cytotoxic capacity are impaired in chronic HIV-1 infection. (A) The expression of GrzA, GrzB, and Prf at resting state was determined in 20 healthy controls and 25 HIV-infected, untreated patients, as well as in 18 paired patient samples before and after effective ART. (B,C) MAIT cell cytotoxic capacity and IFNγ production after 24 h of stimulation with PFA-fixed *E*. *coli* (MOI 10) was determined in 20 healthy controls and 25 HIV-infected, untreated patients, as well as in 18 paired patient samples before and after effective ART as described in (A). Box and whisker plots show median, IQR and the 10^th^ to the 90^th^ percentile. Representative FACS plots are shown. The Mann-Whitney test was used to determine significance between healthy controls and HIV-infected, untreated patients, and the Wilcoxon test for paired samples.

The capacity of MAIT cells to produce pro-inflammatory cytokines is severely decreased in untreated chronic HIV-1 infection [11]. However, it remains unknown whether MAIT cell cytotoxic capacity is also impaired in this condition. To address this, we compared the expression of cytolytic proteins in unstimulated MAIT cells from 20 healthy controls and 25 ART untreated HIV-1 infected patients (Table 1). Levels of GrzA and Prf expressed by MAIT cells in HIV-1 infected patients were modestly, but significantly, lower than those in uninfected healthy controls (Fig 5A, p = 0.0007 and p = 0.0008, respectively). Interestingly, the levels of GrzB expression in MAIT cells from infected patients were significantly higher when compared to healthy controls (p = 0.0004), a pattern in line with the notion that these residual MAIT cells are partially activated, consistent with our previously published data [11]. There was no significant difference in the levels of Gnly in MAIT cells between HIV-1 infected patients and healthy controls (median (IQR) = 16.3 (7.3–28.6)% and 14.5 (7.2–26.0)% respectively; p = 0.98). The modest changes in expression of GrzA, Prf, and GrzB in unstimulated MAIT cells from these patients were not significantly restored following long-term ART (n = 18; Fig 5A) (median (IQR) duration of ART = 38.5 (28–49) months).

**Table 1:** Characteristics of HIV-1 infected patients and healthy controls.

	Stockholm cohort 1			Stockholm cohort 2	
	HIV-	HIV+	p-value	HIV+	p-value
n	20	25		31	
Age, years	43 (33–54)	38 (33–44)	0.13* ^§^	40 (34–49)	0.38^#^ ^§^
Sex, n (%)	14 M (70%)	17 M (68%)	1.0* ^&^	18 M (58%)	0.55^#^ ^&^
CD4 counts, cells/μl	ND	419 (348–496)		340 (253–400)	0.54^#^ ^§^
Viral loads, copies/ml	ND	20500 (2000–66250)		65850 (10198–155500)	0.06^#^ ^§^
Time since HIV diagnosis, months	NA	62 (32–138)		24 (10–69)	0.06^#^ ^§^

ND, not done; NA, not applicable; M, male;

*comparisons were done on uninfected versus HIV-infected patients;

^#^comparisons were done on HIV-infected patients in cohort 1 versus cohort 2;

^§^significance was determined using the Mann-Whitney test;

^&^significance was determined using Fisher’s exact test; median (IQR) is shown for all parameters unless otherwise specified.

Next, we investigated the responses of MAIT cells in terms of GrzB arming and cytolytic potential following a 24 h bacterial stimulation (Fig 5B). All measured facets of MAIT cell cytotoxic potential examined were impaired in chronic HIV-1 infection, including reduction in the levels of MAIT cells expressing CD107a^+^GrzA^lo^ (p = 0.0006), CD107a^+^GrzB^+^ (p<0.0001), and CD107a^+^Prf^+^ (p = 0.0036) phenotypes in response to bacteria (Fig 5B and 5C). The severe deficiency of GrzB-expressing MAIT cells following bacterial stimulation was particularly striking given that this cell population had slightly upregulated GrzB levels at steady state (Fig 5A), indicating that MAIT cells from HIV-1 infected patients have severely compromised ability to upregulate GrzB *de novo* following new microbial encounters. There were no significant correlations between MAIT cell cytotoxic potential and cytokine production with CD4 counts (CD107a^+^GrzA^lo^ Spearman’s r = 0.22, p = 0.32; CD107a^+^GrzB^+^ r = 0.29, p = 0.20; CD107a^+^Prf^+^ r = -0.04, p = 0.88; CD107a^+^IFNγ^+^ r = -0.03, p = 0.91), plasma viral loads (CD107a^+^GrzA^lo^ Spearman’s r = 0.15, p = 0.53; CD107a^+^GrzB^+^ r = 0.02, p = 0.92; CD107a^+^Prf^+^ r = 0.05, p = 0.83; CD107a^+^IFNγ^+^ r = -0.02, p = 0.94), nor with time since HIV diagnosis (CD107a^+^GrzA^lo^ Spearman’s r = -0.17, p = 0.44; CD107a^+^GrzB^+^ r = 0.09, p = 0.69; CD107a^+^Prf^+^ r = -0.26, p = 0.26; CD107a^+^IFNγ^+^ r = -0.22, p = 0.38). Long-term ART partially restored all evaluated aspects of MAIT cell cytotoxic potential (Fig 5C). However, it is important to note that in treated HIV-infected individuals the levels of GrzB up-regulation were still significantly lower (p<0.0001), and levels of Prf tended to be lower (p = 0.063), when compared to healthy controls. There were no significant correlations between the magnitude of MAIT cell cytotoxic capacity and cytokine production recovery after ART with duration of ART (CD107a^+^GrzA^lo^ Spearman’s r = 0.20, p = 0.42; CD107a^+^GrzB^+^ r = 0.14, p = 0.59; CD107a^+^Prf^+^ r = 0.30, p = 0.25; CD107a^+^IFNγ^+^ r = -0.01, p = 0.99).

### Expansion of aberrant MAIT cells lacking T-bet and Eomes in chronic HIV-1 infection correlates with loss of MAIT cells and impaired effector function

**Fig 6:**
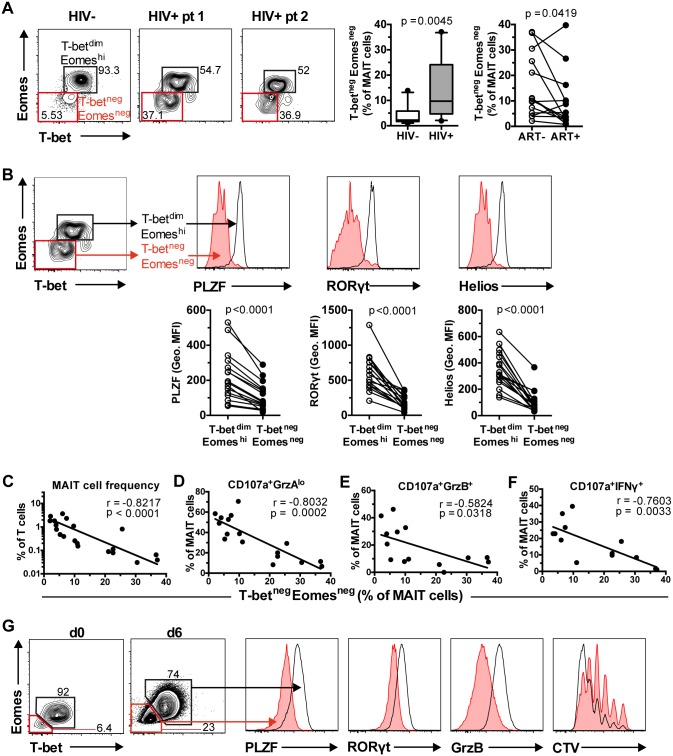
Aberrant MAIT cell transcription factor expression in chronic HIV-1 infection correlates with MAIT cell effector dysfunction. (A) Unstimulated PBMCs from 10 healthy controls and 17 HIV-1 infected, ART-untreated patients were stained for transcription factors, and their MAIT cell T-bet and Eomes expression profile was determined. The effect of long-term ART on MAIT cell T-bet and Eomes expression profile was also determined in paired samples from 14 HIV-infected patients. (B) Comparison of PLZF, RORγt, and Helios levels between the two MAIT cell populations in the same set of HIV-1 infected, ART-untreated patients. (C, D, E, F) Correlations between the levels of T-bet^neg^Eomes^neg^ MAIT cells in ART-untreated HIV-1 infected patients with MAIT cell numbers *ex vivo* and effector functions after *E*. *coli* stimulation (MOI 10) were calculated using Spearman’s test. (G) T-bet^neg^Eomes^neg^ MAIT cells were generated following a six day incubation of PBMCs from healthy controls with PFA-fixed *E*. *coli* (MOI 10). The expression levels of PLZF, RORγt, and GrzB, as well as measurement of proliferation using a Cell Trace Violet (CTV) dilution method, were determined in both T-bet^dim^Eomes^hi^ and T-bet^neg^Eomes^neg^ MAIT cells (n = 3). Box and whisker plot shows median, IQR and the 10^th^ to the 90^th^ percentile. Representative FACS plots are shown. The Mann-Whitney test was used to determine significance between healthy controls and HIV-1 infected, ART-untreated patients, and the Wilcoxon test for paired samples.

In healthy individuals, the vast majority of MAIT cells express low but detectable levels of T-bet and high levels of Eomes (T-bet^dim^Eomes^hi^; Fig 2B and Fig 6A). However, in HIV-1 infected patients there was a significant expansion of a MAIT cell population expressing neither transcription factors at detectable levels (T-bet^neg^Eomes^neg^), and this was only partially reduced following long-term ART (n = 14) (Fig 6A). Detailed investigation revealed that the T-bet^neg^Eomes^neg^ MAIT cell population also expressed significantly lower levels of PLZF, RORγt, and Helios when compared to the T-bet^dim^Eomes^hi^ MAIT cell population (all p<0.0001; Fig 6B). More importantly, the expansion of the T-bet^neg^Eomes^neg^ MAIT cell population in HIV-infected patients was strongly correlated with MAIT cell depletion in the periphery (Fig 6C), loss of MAIT cell cytotoxic potential (CD107a^+^GrzA^lo^ and CD107a^+^GrzB^+^; Fig 6D and 6E, respectively), and impaired IFNγ production (Fig 6F) in response to bacterial stimulation.

Next, we investigated whether this aberrant MAIT cell transcription factor profile could be generated *in vitro* following a strong chronic antigenic exposure. A six day culture of PBMCs from healthy donors (n = 3) with mildly fixed *E*. *coli* increased the frequency of T-bet^neg^Eomes^neg^ MAIT cells (Fig 6G). Similar to MAIT cells in HIV-infected patients with this phenotype, these *in vitro*-generated T-bet^neg^Eomes^neg^ MAIT cells also expressed lower levels of PLZF and RORγt (Fig 6G). The T-bet^neg^Eomes^neg^ MAIT cells also expressed lower levels of GrzB. Finally, the aberrant T-bet^neg^Eomes^neg^ MAIT cells proliferated less when compared to T-bet^dim^Eomes^hi^ MAIT cells (Fig 6G).

### IL-7 significantly restores the impaired MAIT cell effector function, and correlates with MAIT cell numbers in HIV-1 infected patients

**Fig 7:**
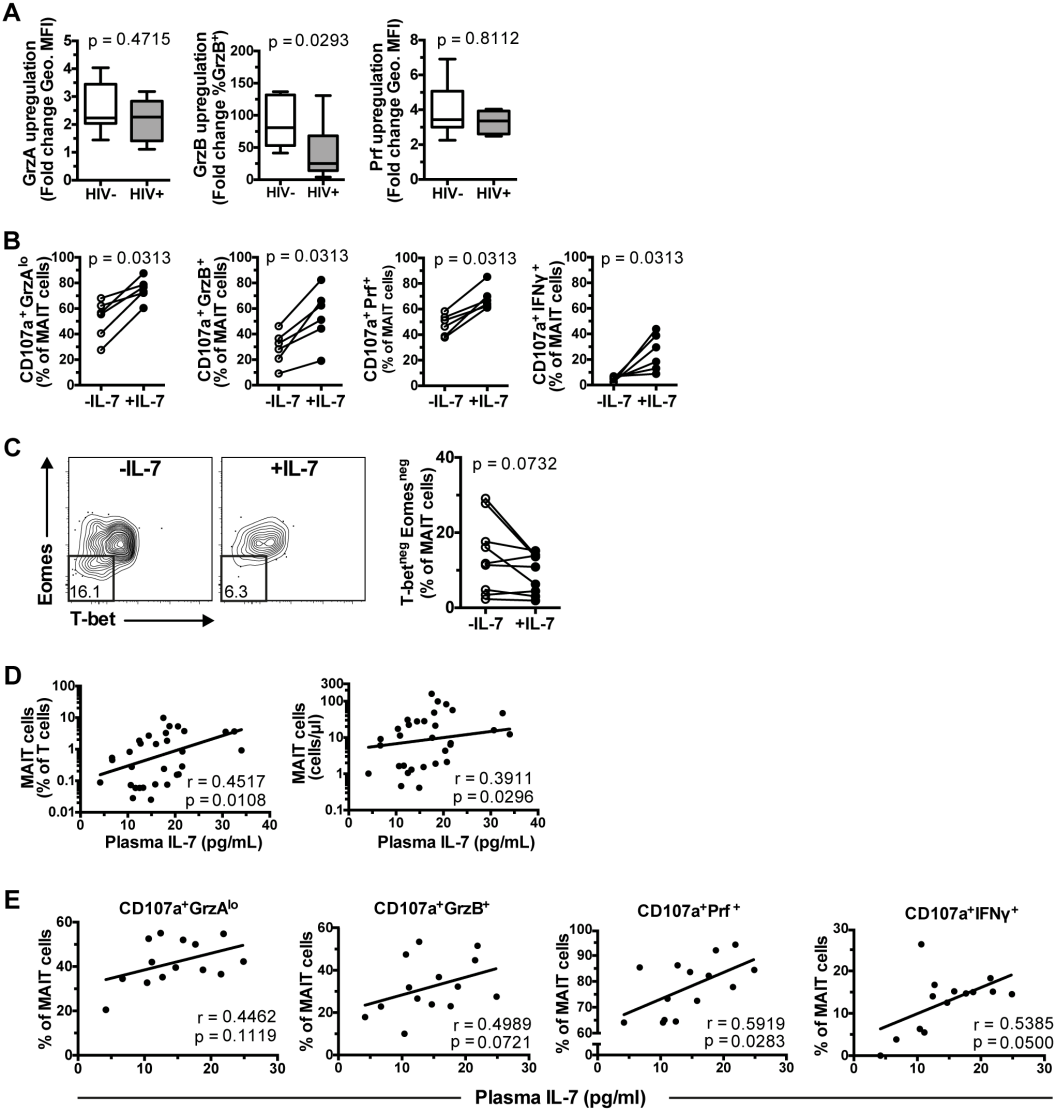
IL-7 restores MAIT cell effector dysfunction. (A) MAIT cell arming following IL-7 treatment *in vitro* for 48 h in healthy controls (n = 8), and HIV-1 infected ART-untreated patients (n = 6). GrzA and Prf upregulation was determined as fold-change of geometric MFI to that of IL-7-untreated cells, whereas GrzB upregulation was determined as fold change of the percentage GrzB-expressing cells. (B) PBMCs from six HIV-1 infected ART-untreated patients were either left untreated or treated with IL-7 for 24 h, then stimulated with mildly PFA-fixed *E*. *coli* (MOI 10) for a further 24 h before measurements of MAIT cell effector functions. (C) PBMCs from nine HIV-1 infected ART-untreated patients were incubated in the presence or absence of 10 ng/ml IL-7 for 48 h and then stained for transcription factor expression as described in Fig 6. Correlation between plasma levels of IL-7 and MAIT cell frequency and numbers from 31 HIV-1 infected ART-untreated patients (D) or MAIT cell effector function from 14 patients (E) were calculated using Spearman’s test. Box and whisker plots show median, IQR and the 10^th^ to the 90^th^ percentile. Representative FACS plots are shown. Significance between independent samples was determined using Mann-Whitney’s test, and paired samples using Wilcoxon’s signed rank test (B) or paired t-test (C).

The potential of IL-7 treatment to restore MAIT cell functional defects in HIV-1 infected patients was evaluated *in vitro*. Interestingly, GrzA and Prf upregulation following IL-7 treatment in samples from healthy controls (n = 8) and HIV-1 infected patients (n = 6) was not significantly different (Fig 7A). However, whereas GrzB upregulation occurred in MAIT cells from both groups, it was weaker in HIV-1 infected patients suggesting that the cytotoxicity arming effect of IL-7 in these patients may be less distinct (p = 0.0293, Fig 7A). In a second round of experiments the ability of IL-7 to support the MAIT cell response in HIV-1 infected patients to a 24 h bacterial stimulation was evaluated. IL-7 treatment significantly improved all measured phenotypic aspects of MAIT cell cytotoxic potential including the generation of CD107a^+^GrzA^lo^, CD107a^+^GrzB^+^, and CD107a^+^Prf^+^ phenotypes, as well as the production of IFNγ by MAIT cells (n = 6) (Fig 7B). Next, we investigated whether the improvement of MAIT cell effector functions by IL-7 treatment might be linked with restoration of the abnormal MAIT cell transcription factor profile in nine ART-untreated HIV-1 infected patients. Importantly there was no difference in CD127 levels by T-bet^neg^Eomes^neg^ and T-bet^dim^Eomes^hi^ MAIT cells (S4A Fig). Next, PBMCs were cultured with IL-7 for a maximum of 48 h to minimize introducing potential confounders, including global cellular proliferation, activation-induced cell death, and HIV-1 replication that may result from a long-term IL-7 exposure. The frequency of T-bet^neg^Eomes^neg^ MAIT cells showed a trend towards a decrease during this culture (n = 9, p = 0.0732; Fig 7C). Short-term IL-7 treatment also reduced the already low frequency of T-bet^neg^Eomes^neg^ MAIT cells in seven HIV-uninfected healthy controls (p = 0.0156; S4B Fig).

We next evaluated whether plasma levels of IL-7 *in vivo* were associated with MAIT cell levels in 31 individuals enrolled in a second cohort of HIV-infected patients recruited from the same site (Table 1). The clinical parameters of cohort 2 were not significantly different when compared to those of cohort 1. Furthermore, patients in cohort 2 saw a similarly significant depletion of MAIT cells, and a similarly significant increase in the levels of Vα7.2^+^CD161^-^ T cells in peripheral blood (S5A Fig) as previously reported [11]. There was also a significant increase in MAIT cell activation as evaluated by expression of CD38, HLA-DR, CD57, and TIM-3 (S5B Fig), and an inverse correlation between the levels of MAIT cells in circulation and MAIT cell CD38 expression (S5C Fig). Consistent with our previous study and other studies [11, 21, 29, 31], there were no significant correlations between MAIT cell levels and either CD4 counts and plasma viral loads (Spearman’s r = 0.074, p = 0.70; and r = 0.22, p = 0.23, respectively). We also did not find any correlation between MAIT cell levels and plasma markers of microbial translocation (LPS; Spearman’s r = 0.018, p = 0.93) and innate immune activation (sCD14; r = -0.14, p = 0.45).

Having thus validated that cohort 2 was comparable to cohort 1, the patients in cohort 2 displayed a positive correlation between the plasma levels of IL-7 and MAIT cell frequency and absolute counts in peripheral blood (r = 0.45, p = 0.011, and r = 0.39, p = 0.030, respectively; Fig 7D). Furthermore, plasma IL-7 levels showed a weak positive correlation with the capacity of MAIT cells to respond to PFA-fixed *E*. *coli* stimulation (CD107a^+^GrzA^lo^ Spearman’s r = 0.45, p = 0.11; CD107a^+^GrzB^+^ r = 0.50, p = 0.072; CD107a^+^Prf^+^ r = 0.59, p = 0.028; CD107a^+^IFNγ^+^ r = 0.54, p = 0.050) (Fig 7E). While there were weak correlations with CD4 counts (Spearman’s r = -0.34, p = 0.017) and plasma viral loads (r = 0.29, p = 0.040), there were no significant relationships between plasma IL-7 levels and CD38, HLA-DR, CD57, and TIM-3 expression in MAIT cells (S6 Fig). Taken together, these results suggest that IL-7 plasma levels may directly influence MAIT cell numbers and their capacity to respond to microbial challenge in HIV-infected patients.

## Discussion

MAIT cells are emerging as a significant component of the cellular immune defenses against microbial infection. They are found in circulation and enriched in intestinal mucosa, liver and lung. We and others have shown that patients with HIV-1 infection suffer numerical loss and functional decline of their MAIT cells. This was at first glance unexpected given that MAIT cells are mostly CD8^+^ or DN T cells with only very few expressing CD4. Furthermore, the antigens recognized by MAIT cells are of bacterial and fungal origin meaning that the exhausted functional phenotype of MAIT cells may be due to engagement not in antiviral immune responses, but rather in a response against microbes at mucosal barriers. Here, we have shown that the functional defect in MAIT cells also includes the cytolytic potential, with low levels of GrzA and Prf expression and a particularly striking defect in GrzB arming. This is likely to have significant consequences for MAIT cell-mediated control of intracellular microbes where direct cytolysis plays a significant role. MAIT cells play a role in immune defense against mycobacteria [8, 33], are involved in tuberculosis [40], as well as sepsis and severe bacterial infections in humans [35]. Even in the current era of effective ART, HIV-1 infected patients have impaired control of mycobacterial and other non-opportunistic pathogens, with increased risk of developing infections from such pathogens [27]. Bacterial sepsis is now the principal cause of intensive care unit admission and death for HIV-1-infected patients who are admitted to hospital even in western countries [41]. Because of MAIT cells’ abundance, strategic locations at the body barrier sites, and potent antimicrobial activities against diverse pathogenic microbes, the broad numerical decline and severe dysfunction of MAIT cells may significantly contribute to morbidities and mortalities from both AIDS-defining infections and non-AIDS-related infections in HIV-1-infected patients. Importantly, long-term effective ART did not rescue the ability of MAIT cells to arm with GrzB in response to bacterial antigen, indicating that this deficiency may be largely irreversible in HIV-infected people.

The development and function of MAIT cells is dependent on expression of the transcription factor PLZF, and we also confirm that they express RORγt consistent with their potential to produce IL-17. In fact, our data suggest that the combination of PLZF and RORγt expression is sufficiently distinctive such that it can be used to identify this innate T cell population in the absence of the Vα7.2 TCR and CD161 combination. In addition, MAIT cells express the two classical effector T cell T-box transcription factors T-bet and Eomes, as well as Helios, with low expression levels for T-bet, high levels for Eomes, and intermediate levels for Helios. This pattern is in keeping with the effector memory-like phenotype of MAIT cells [42, 43]. Helios is a member of the Ikaros family and is predominantly studied in relation to regulatory T (Treg) cell subsets [44, 45]. Recently, however, the role of Helios beyond Treg subsets has been recognized, including as a marker for T cell activation, proliferation, and helper T cell subsets [46, 47]. The role of Helios in MAIT cells is currently unclear and warrants further studies. Nevertheless, this pattern indicates that MAIT cells express a unique combination of these critical transcription factors and may have a unique transcriptional program underlying their distinct phenotypic and functional profile. Interestingly, the exhausted characteristics of MAIT cells in HIV-1 infected patients were paired with the appearance of a dysregulated expression pattern of these critical transcription factors. The occurrence of MAIT cells deficient in both Eomes and T-bet correlates strongly with both the functional impairment and the numerical decline of these cells. Of note, such aberrant MAIT cells also had low levels of RORγt, which is in line with our previous observation that MAIT cells from HIV-infected patients were unable to produce IL-17 following bacterial stimulation [11]. This indicates that the defects of MAIT cells are broad-based and occur at the transcriptional level. It is possible that this effect might be due to continuous antigenic burden relating to loss of control of bacterial infections as well as to microbial translocation in HIV-infected patients. Indeed, chronic stimulation with mildly fixed *E*. *coli* generated MAIT cells deficient in both T-bet and Eomes. Importantly, these *in vitro*-generated MAIT cells lacking T-bet and Eomes also had low GrzB levels, consistent with the partially redundant activities of T-bet and Eomes in inducing GrzB in cytotoxic CD8 T cells [48, 49]. It is however possible that other pathways may be involved in the generation of T-bet and Eomes defective MAIT cells *in vivo*.

IL-7 has strong effects on T cell homeostasis and supports the survival of T cells by upregulating Bcl-2. These characteristics have made IL-7 attractive for cytokine immunotherapy in humans. MAIT cells express high levels of the IL-7Rα (CD127) [12, 38], a finding that opened the possibility that IL-7 may have strong effects on MAIT cells. Interestingly, IL-7 alone in the absence of other stimuli was capable of arming resting MAIT cells from healthy donors into cytotoxic GrzB+ effector T cells. The induction of GrzB is profound, as resting MAIT cells essentially lack expression of this protein. Along with this effect, MAIT cells also further upregulated Prf and GrzA, without inducing any detectable spontaneous cytokine production. In the absence of bacterial antigen the effect of IL-7 is thus specifically to induce the cytotoxic arming of MAIT cells. Importantly, this in turn leads to a significantly increased cytolytic activity against MR1-expressing targets pulsed with bacteria. Perhaps an even more striking and potentially physiologically important effect is the radically enhanced sensitivity of MAIT cells to very low bacterial doses after IL-7 treatment. IL-7 supports strong cytokine production and cytolytic responses at antigen doses that are otherwise non-stimulatory for MAIT cells. These effects are consistent with the observations by Tang et al., where IL-7 enhanced expression of the TCR, CD8 chains and components of TCR signaling pathway [38]. Altogether, IL-7 may be suitable for immunotherapy approaches aimed at enhancing MAIT cell function in humans.

MAIT cells from HIV-infected patients significantly restored their effector functions after a short incubation with IL-7 *in vitro*. This effect was evident both in terms of arming, i.e. upregulation of in particular GrzB in resting non-antigen activated MAIT cells, and in terms of boosting the anti-bacterial degranulation and IFNγ response in MAIT cells. It should however be noted that the low levels of residual MAIT cells did not allow direct assessment of MAIT cell cytolytic capacity in HIV-1 infected patients. Interestingly, IL-7 has received attention as an adjunctive cytokine immunotherapy in HIV-1 infected patients to help restore immune functions that remain impaired even after successful ART. Particularly interesting were the recent observations by Sereti et al., that IL-7 treatment helped replenish the mucosal T cell compartment and supported an improved mucosal barrier function as evidenced by decreases in relevant biomarkers of microbial translocation [50]. Our findings in the present study open the possibility that these effects in patients involve the MAIT cell compartment. In such a model the mucosal T cell subsets including MAIT cells improve their numbers and function to alleviate the gut mucosal abnormalities of chronic HIV-1 infection. This possibility is further supported by the weak positive correlation we observe between plasma IL-7 levels and MAIT cell levels *in vivo*.

In summary, the findings in this study indicate that arming of cytolytic capacity occurs rapidly upon detection of bacterial antigen in MAIT cells in healthy donors, and this response is severely defective in HIV-1 infected patients. The broad-based functional defects in MAIT cells in chronic HIV-1 infection are associated with deficiency in the critical transcription factors Eomes and T-bet. Furthermore, our data show that IL-7 induces the arming of effector functions and enhances the sensitivity of MAIT cells in healthy donors, and can partially reverse the functional and transcription factor defects in MAIT cells from HIV-1 infected patients. These findings support the inclusion of IL-7 in immunotherapeutic approaches to restore MAIT cell numbers and function in HIV-1 infection as well as other conditions associated with loss and dysfunction of these cells.

## Materials and Methods

### Ethics statement

Written informed consent was obtained from all study participants in accordance with study protocols conforming to the provisions of the Declaration of Helsinki and approved by the Regional Ethics Review Board in Stockholm (Protocols 2007/772-32, 401/01, and 2009-1485-31-3).

### Study participants

HIV-1 infected patients were from the Karolinska University Hospital Huddinge Infectious Diseases Outpatient Clinic (Stockholm, Sweden) (Table 1) [11]. Inclusion criteria were that patients were HIV-1 seropositive and had no history of AIDS-defining illness in the 12 months prior to recruitment. Healthy HIV-uninfected individuals were recruited at the Blood Transfusion Clinic at the Karolinska University Hospital Huddinge.

### Peripheral blood processing

PBMCs were isolated from peripheral blood by Ficoll-Hypaque density gradient centrifugation (Pfizer-Pharmacia or Axis-Shield), and either allowed to rest overnight in complete medium, or cryopreserved in liquid nitrogen until required.

### Functional assay

PBMCs were cultured in 10 ng/ml recombinant human IL-7 (R&D Systems), a combination of IL-12 and IL-18 (10 ng/ml and 100 ng/ml, respectively; PeproTech) for 24–48 h, as indicated or left untreated at 37°C and 5% CO_2_ in RPMI medium supplemented with 10% fetal calf serum and 50 μg/ml gentamicin (Gibco) (RF10 medium) prior to functional assay. MAIT cell functions were determined *in vitro* using a paraformaldehyde (PFA)-fixed *E*. *coli* stimulation (D21 strain, MOI as indicated) in the presence of 1.25 μg/ml anti-CD28 mAb (BD Biosciences) [11]. PBMCs were further cultured for 24 h, and in selected experiments, 0.4 μg/ml anti-CD107a PECy7 (BD Biosciences) was added at the start of bacterial stimulation, and monensin (Golgi Stop, BD Biosciences) was added during the last six hours of the stimulation. In selected experiments, cells were stained with Cell Trace Violet (CTV) Cell Proliferation Kit (Life Technologies) as per manufacturer’s instructions and cultured in RF10 medium with fixed *E*. *coli* in the presence of anti-CD28 for six days as described [12].

### Cytotoxicity assay

Vα7.2^+^ T cells were purified from freshly isolated PBMCs using Vα7.2-APC antibody, followed by anti-APC microbeads (Miltenyi Biotec) and positive selection using MACS Cell Separation (Miltenyi Biotec). The purity of enriched Vα7.2^+^ T cells was typically >95%, with minimum MAIT cell purity of 90%. Vα7.2^+^ T cells were cultured in RF10 medium supplemented with 25 ng/ml recombinant human IL-7 or in RF10 medium alone for 72 h. Human 293T cells stably transfected with human MR1 (293T-hMR1 cells; a kind gift from Dr. Ted Hansen) [4] were incubated with PFA-fixed *E*. *coli* at an MOI of 10 for three hours, followed by the addition of anti-MR1 mAb (26.5; Biolegend) or IgG2a isotype control (MOPC-173; Biolegend) for 60 min prior to the addition of Vα7.2^+^ T effector cells at the indicated effector to target (MAIT:293T-hMR1) ratio. The MAIT:293T-hMR1 ratio was adjusted to take into accounts the overall purity of MAIT cells within the Vα7.2^+^ T cell population as the contaminating Vα7.2^+^CD161^-^ T cells did not mediate cytotoxicity. Target cell apoptosis was detected through the fluorescent inhibitor of caspases (FLICA) flow cytometry-based methodology. Briefly, the FLICA reagent (Vybrant FAM Poly Caspases Assay Kit, Life Technologies) was added at a final concentration of 0.2% (v/v) to the MAIT-293T-hMR1 cell culture at the beginning of the assay, and anti-CD107a PECy7 at a total concentration of 0.4 μg/ml was also added to detect MAIT cell degranulation. After 24 h of culture, cells were harvested and stained to simultaneously detect MAIT cell cytotoxicity and 293T-hMR1 cell death.

### Antibodies, flow cytometry and IL-7 measurement

Anti-CD3 Alexa Fluor (AF)700 (clone UCHT1), anti-CD4 APC-H7 (clone SK3), anti-CD38 PECy7 (clone HIT2), anti-CD161 PECy5 (clone DX12), anti-GrzB AF700 (clone GB11), anti-HLA-DR APC-H7 (clone L243), anti-RORγt PE (clone Q21-559), anti-TNF PECy7 (clone MAb11) were from BD Biosciences; anti-CD3 Brilliant Violet (BV)510 and BV785 (clone OKT3), anti-CD4 BV711 (clone OKT4), anti-CD8 BV570 (clone RPA-T8), anti-CD57 Pacific Blue, anti-CD107a PECy7 (clone H4A3), anti-CD127 BV650 (clone A019D5), anti-CD161 BV605 (clone HP-3G10), anti-granulysin PE (clone DH2), anti-granzyme (Grz)A AF700 (clone CB9), anti-GrzB FITC (clone GB11), anti-IFNγ BV785 (clone 4S.B3), anti-IL-17A BV421 and BV711 (clone BL168), anti-Ki67 BV421 (clone Ki-67), anti-perforin BV421 (clone B-D48), anti-T-bet BV605 and BV711 (clone 4B10), and anti-Vα7.2 APC, PE, and PECy7 (clone 3C10) were from Biolegend; anti-Eomes FITC (clone WD1928), and anti-Helios eFluor 450 (clone 22F6) were from Ebioscience; anti-CD8 Q-dot 655 (clone 3B5), and live/dead aqua and near infrared fixable cell stain were from Life Technologies; anti-PLZF APC (clone 6318100), and anti-TIM-3 AF488 (clone 344823) were from R&D systems.

Cell surface staining was performed using directly conjugated antibodies and fixed in Cytofix/Cytoperm or in Transcription Factor Fixation/Permeabilization buffer (both from BD Biosciences) as appropriate. Intracellular staining was performed using the relevant mAbs in Perm/Wash or Transcription Factor Perm/Wash buffer as appropriate (both from BD Biosciences). Samples were acquired on an LSRFortessa 18-colour flow cytometer (BD Biosciences) equipped with 405, 488, 561 and 639 nm lasers. Single-stained polystyrene beads (BD Biosciences) were used for compensation purposes. Software-based compensation was performed using the compensation platform in FlowJo software version 9.6 (Tree Star).

Circulating IL-7 was detected in plasma (diluted 1:4) using the Quantikine HS Human IL-7 Assay (RnD Systems, Abingdon, UK), according to the manufacturer’s instructions. Each sample was assayed in duplicate.

### Statistical analysis

Significant differences in independent samples were assessed using Fisher’s exact test for categorical variables. Continuous variables were first assessed for normality, and differences in independent samples were assessed using t-test or Mann-Whitney test for continuous variables as appropriate. The Wilcoxon signed rank test or paired t-test as appropriate was used to determine significance between paired samples. The Friedman test followed by Dunn’s post-hoc test was used to detect differences across multiple, paired samples. Correlations were evaluated using Spearman’s rank correlation. Statistical analyses were performed on raw data using Prism version 6.0f (GraphPad), and two-sided p-values < 0.05 were considered significant.

## Supporting Information

S1 Fig Cytolytic protein and transcription factor expression profiles in MAIT cells. (A) Most of MAIT cell cytolytic proteins expressed at steady state are co-expressed with perforin (Prf). FACS plots representative of data from 20 healthy individuals are shown. (B) PLZF and RORγt co-expression in the CD8^+^ T cell population sufficiently identifies the MAIT cell population. Freshly isolated PBMCs from 10 healthy individuals were stained for the transcription factors PLZF and RORγt. The co-expression patterns for these transcription factors, PLZF^+^ RORγt^+^ (gate no. 1), PLZF^-^ RORγt^+^ (gate no. 2), PLZF^+^ RORγt^-^ (gate no. 3) were determined in total T cells, CD8^+^ T cells, and CD4^-^ CD8^-^ double-negative (DN) T cells. MAIT cells were identified from these sub-populations by their Vα7.2 TCR and high levels of CD161 co-expression. Box and whisker plot shows median, IQR and the 10^th^ to the 90^th^ percentile. Significance across multiple paired samples was determined by the Friedman test followed by Dunn’s post-hoc test. Representative FACS plots from a single individual are shown.
(PDF)

S2 Fig Dose-response curve and MR1-independency of IL-7 arming of MAIT cell cytotoxicity. Freshly isolated PBMCs from three healthy donors were incubated with a range of IL-7 concentrations as indicated or left untreated for 24 h. Cells were then cultured for another 24 h in the absence (A, B, C), or presence of PFA-fixed *E*. *coli* (MOI 10) (D), before staining for cytolytic proteins and IFNγ. (B) To determine the MR1-dependency of IL-7 arming of MAIT cell cytotoxicity, freshly isolated PBMCs were incubated with 10 ng/ml IL-7 in the presence of anti-MR1 or IgG2a isotype control for 24 h. Cells were then harvested and stained for cytolytic proteins and IFNγ. Representative FACS plots from two independent donors are shown. Error bars represent mean and standard deviation.
(PDF)

S3 Fig Arming of MAIT cell cytotoxicity by IL-7 compared to activation by IL-12 and IL-18. (A) Freshly isolated PBMCs from three healthy donors were incubated with 10 ng/ml of IL-7, a combination of IL-12 and IL-18 (10 ng/ml and 100 ng/ml, respectively), or left untreated for 48 h. Cells were further cultured for the next 24 h in the absence or presence of PFA-fixed *E*. *coli* stimulation (MOI 10) (A and B, respectively) before staining for MAIT cell cytolytic proteins and IFNγ. (C) MAIT cell frequency following *E*. *coli* stimulation in the presence or absence of IL-7, or in a combination of IL-12 and IL-18, was determined in three healthy individuals (left panel) or in eight healthy individuals (right panel). The Friedman test followed by Dunn’s post-hoc test was used to determine significance across multiple, paired samples. Error bars represent median and IQR, and box and whisker plot shows median, IQR and the 10^th^ to the 90^th^ percentile. (D) Spearman rank correlation between the capacity of healthy donor (n = 18) MAIT cells to upregulate GrzB and degranulate following a 24 h co-culture with *E*. *coli*-pulsed 293T-hMR1 cells, and the 293T-hMR1 cell death.
(PDF)

S4 Fig The influence of a short-course IL-7 treatment *in vitro* on T-bet^neg^ Eomes^neg^ MAIT cell levels. (A) The levels of CD127 expression in T-bet^dim^ Eomes^hi^ and T-bet^neg^ Eomes^neg^ MAIT cells from nine HIV-1 infected ART-untreated patients. (B) PBMCs from seven healthy controls were incubated with 10 ng/ml of IL-7 for 48 h and then stained for transcription factor expression as described in Fig 6. Significance was determined using the paired t-test.
(PDF)

S5 Fig MAIT cell depletion in chronically HIV-1 infected patient cohort 2 is associated with activation and exhaustion phenotypes, as previously observed for cohort 1. (A) The frequency of MAIT cells and Vα7.2^+^ CD161^-^ T cells from 20 healthy controls and 31 untreated HIV-infected patients. (B) The expression of CD38, HLA-DR, CD57, and TIM-3 was then determined on the MAIT cell population from these individuals. Significance was determined using the Mann-Whitney test. Box and whisker plots show median, IQR and the 10^th^ to the 90^th^ percentile. (C) Spearman’s rank correlation between CD38^hi^-expressing MAIT cells and the frequency of MAIT cells in the peripheral blood of 31 untreated HIV-infected patients.
(PDF)

S6 Fig Plasma IL-7 levels weakly correlate with CD4 counts and plasma viral load, but not with MAIT cell activation markers. Relationships between plasma IL-7 levels and CD4 counts (A), plasma viral loads (B), as well as with the MAIT cell activation markers CD38 (C), HLA-DR (D), CD57 (E), and TIM-3 (F) was assessed using Spearman’s rank correlation in 31 ART-untreated HIV-infected patients.
(PDF)
