# Supplementary material for: Arming of MAIT Cell Cytolytic Antimicrobial Activity Is Induced by IL-7 and Defective in HIV-1 Infection
Source: PLoS Pathog. 2015 Aug 21;11(8):e1005072. doi: 10.1371/journal.ppat.1005072 (PMC4546682; doi:10.1371/journal.ppat.1005072)
Supplement: S1 Fig — (A) Most of MAIT cell cytolytic proteins expressed at steady state are co-expressed with perforin (Prf). FACS plots representative of data from 20 healthy individuals are shown. (B) PLZF and RORγt co-expression in the CD8+ T cell population sufficiently identifies the MAIT cell population. Freshly isolated PBMCs from 10 healthy individuals were stained for the transcription factors PLZF and RORγt. The co-expression patterns for these transcription factors, PLZF+ RORγt+ (gate no. 1), PLZF- RORγt+ (gate no. 2), PLZF+ RORγt- (gate no. 3) were determined in total T cells, CD8+ T cells, and CD4- CD8- double-negative (DN) T cells. MAIT cells were identified from these sub-populations by their Vα7.2 TCR and high levels of CD161 co-expression. Box and whisker plot shows median, IQR and the 10th to the 90th percentile. Significance across multiple paired samples was determined by the Friedman test followed by Dunn’s post-hoc test. Representative FACS plots from a single individual are shown. (PDF) [file ppat.1005072.s001.pdf]

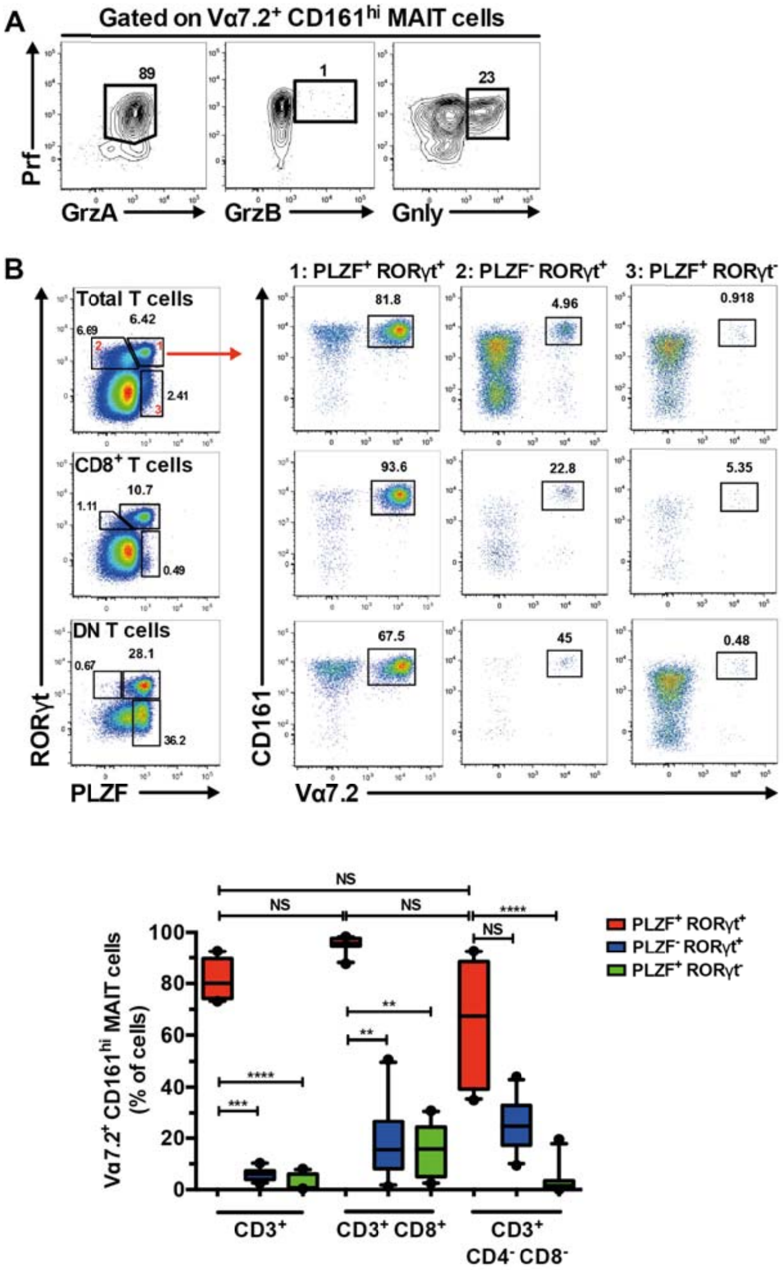

S1 Fig. Cytolytic protein and transcription factor expression profiles in MAIT cells. (A) Most of MAIT cell cytolytic proteins expressed at steady state are co-expressed with perforin (Prf). FACS plots representative of data from 20 healthy individuals are shown. (B) PLZF and RORγt co-expression in the $CD8^+$ T cell population sufficiently identifies the MAIT cell population. Freshly isolated PBMCs from 10 healthy individuals were stained for the transcription factors PLZF and RORγt. The co-expression patterns for these transcription factors, $PLZF^+$ $ROR\gamma t^+$ (gate no. 1), $PLZF^-$ $ROR\gamma t^+$ (gate no. 2), $PLZF^+$ $ROR\gamma t^-$ (gate no. 3) were determined in total T cells, $CD8^+$ T cells, and $CD4^-$ $CD8^-$ double-negative (DN) T cells. MAIT cells were identified from these sub-populations by their Vα7.2 TCR and high levels of CD161 co-expression. Box and whisker plot shows median, IQR and the 10th to the 90th percentile. Significance across multiple paired samples was determined by the Friedman test followed by Dunn's post-hoc test. Representative FACS plots from a single individual are shown.
