# Supplementary material for: Arming of MAIT Cell Cytolytic Antimicrobial Activity Is Induced by IL-7 and Defective in HIV-1 Infection
Source: PLoS Pathog. 2015 Aug 21;11(8):e1005072. doi: 10.1371/journal.ppat.1005072 (PMC4546682; doi:10.1371/journal.ppat.1005072)
Supplement: S2 Fig — Freshly isolated PBMCs from three healthy donors were incubated with a range of IL-7 concentrations as indicated or left untreated for 24 h. Cells were then cultured for another 24 h in the absence (A, B, C), or presence of PFA-fixed E. coli (MOI 10) (D), before staining for cytolytic proteins and IFNγ. (B) To determine the MR1-dependency of IL-7 arming of MAIT cell cytotoxicity, freshly isolated PBMCs were incubated with 10 ng/ml IL-7 in the presence of anti-MR1 or IgG2a isotype control for 24 h. Cells were then harvested and stained for cytolytic proteins and IFNγ. Representative FACS plots from two independent donors are shown. Error bars represent mean and standard deviation. (PDF) [file ppat.1005072.s002.pdf]

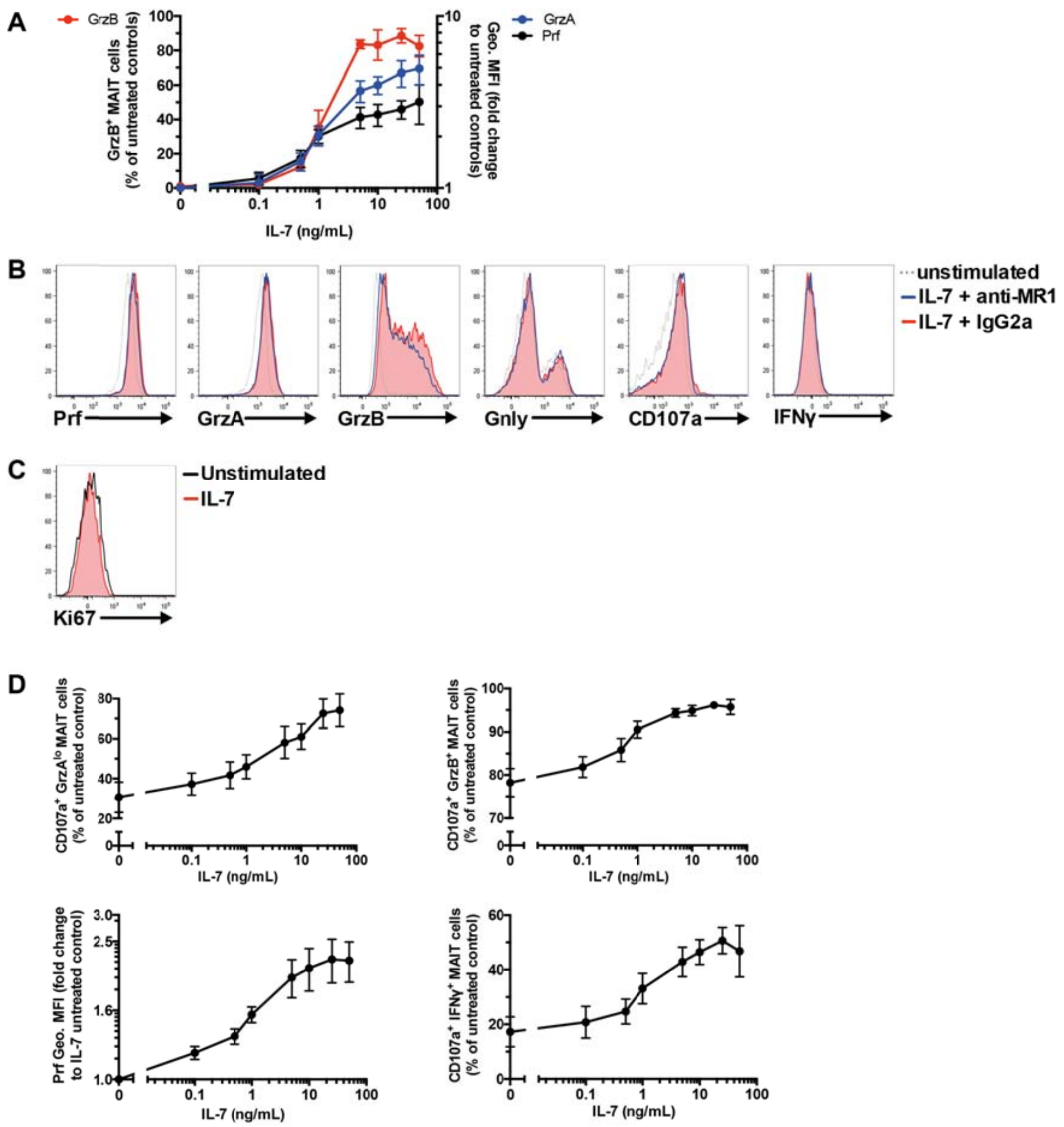

S2 Fig. Dose-response curve and MR1-independency of IL-7 arming of MAIT cell cytotoxicity. Freshly isolated PBMCs from three healthy donors were incubated with a range of IL-7 concentrations as indicated or left untreated for 24 h. Cells were then cultured for another 24 h in the absence (A, B, C), or presence of PFA-fixed *E. coli* (MOI 10) (D), before staining for cytolytic proteins and IFNγ. (B) To determine the MR1-dependency of IL-7 arming of MAIT cell cytotoxicity, freshly isolated PBMCs were incubated with 10 ng/ml IL-7 in the presence of anti-MR1 or IgG2a isotype control for 24 h. Cells were then harvested and stained for cytolytic proteins and IFNγ. Representative FACS plots from two independent donors are shown. Error bars represent mean and standard deviation.
