# Supplementary material for: Arming of MAIT Cell Cytolytic Antimicrobial Activity Is Induced by IL-7 and Defective in HIV-1 Infection
Source: PLoS Pathog. 2015 Aug 21;11(8):e1005072. doi: 10.1371/journal.ppat.1005072 (PMC4546682; doi:10.1371/journal.ppat.1005072)
Supplement: S3 Fig — (A) Freshly isolated PBMCs from three healthy donors were incubated with 10 ng/ml of IL-7, a combination of IL-12 and IL-18 (10 ng/ml and 100 ng/ml, respectively), or left untreated for 48 h. Cells were further cultured for the next 24 h in the absence or presence of PFA-fixed E. coli stimulation (MOI 10) (A and B, respectively) before staining for MAIT cell cytolytic proteins and IFNγ. (C) MAIT cell frequency following E. coli stimulation in the presence or absence of IL-7, or in a combination of IL-12 and IL-18, was determined in three healthy individuals (left panel) or in eight healthy individuals (right panel). The Friedman test followed by Dunn’s post-hoc test was used to determine significance across multiple, paired samples. Error bars represent median and IQR, and box and whisker plot shows median, IQR and the 10th to the 90th percentile. (D) Spearman rank correlation between the capacity of healthy donor (n = 18) MAIT cells to upregulate GrzB and degranulate following a 24 h co-culture with E. coli-pulsed 293T-hMR1 cells, and the 293T-hMR1 cell death. (PDF) [file ppat.1005072.s003.pdf]

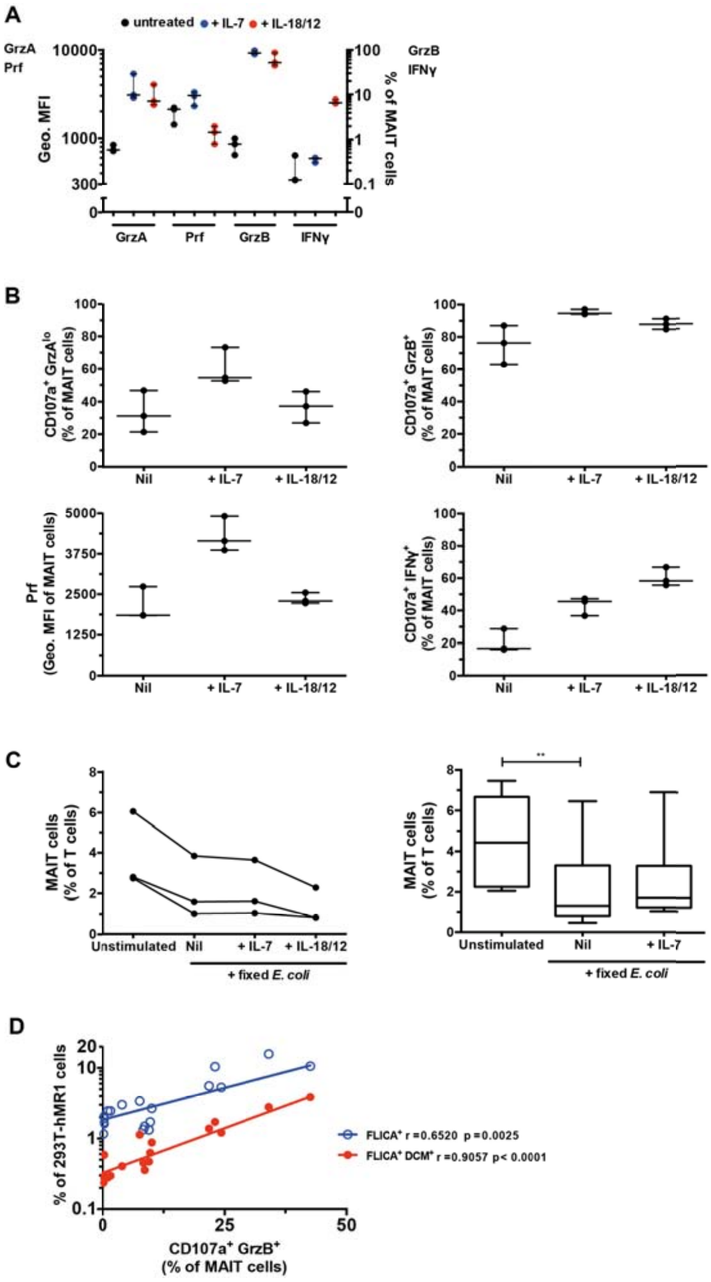

S3 Fig. Arming of MAIT cell cytotoxicity by IL-7 compared to activation by IL-12 and IL-18. (A) Freshly isolated PBMCs from three healthy donors were incubated with 10 ng/ml of IL-7, a combination of IL-12 and IL-18 (10 ng/ml and 100 ng/ml, respectively), or left untreated for 48 h. Cells were further cultured for the next 24 h in the absence or presence of PFA-fixed *E. coli* stimulation (MOI 10) (A and B, respectively) before staining for MAIT cell cytolytic proteins and IFNγ. (C) MAIT cell frequency following *E. coli* stimulation in the presence or absence of IL-7, or in a combination of IL-12 and IL-18, was determined in three healthy individuals (left panel) or in eight healthy individuals (right panel). The Friedman test followed by Dunn's post-hoc test was used to determine significance across multiple, paired samples. Error bars represent median and IQR, and box and whisker plot shows median, IQR and the 10th to the 90th percentile. (D) Spearman rank correlation between the capacity of healthy donor (n=18) MAIT cells to upregulate GrzB and degranulate following a 24 h co-culture with *E. coli*-pulsed 293T-hMR1 cells, and the 293T-hMR1 cell death.
