# Supplementary material for: Arming of MAIT Cell Cytolytic Antimicrobial Activity Is Induced by IL-7 and Defective in HIV-1 Infection
Source: PLoS Pathog. 2015 Aug 21;11(8):e1005072. doi: 10.1371/journal.ppat.1005072 (PMC4546682; doi:10.1371/journal.ppat.1005072)
Supplement: S4 Fig — (A) The levels of CD127 expression in T-betdim Eomeshi and T-betneg Eomesneg MAIT cells from nine HIV-1 infected ART-untreated patients. (B) PBMCs from seven healthy controls were incubated with 10 ng/ml of IL-7 for 48 h and then stained for transcription factor expression as described in Fig 6. Significance was determined using the paired t-test. (PDF) [file ppat.1005072.s004.pdf]

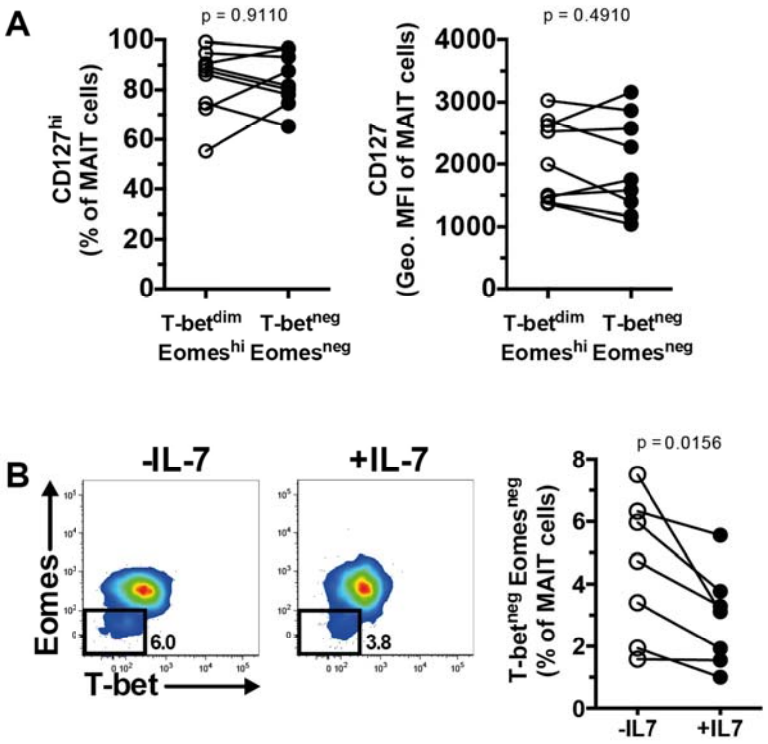

S4 Fig. The influence of a short-course IL-7 treatment *in vitro* on T-bet^{neg} Eomes^{neg} MAIT cell levels. (A) The levels of CD127 expression in T-bet^{dim} Eomes^{hi} and T-bet^{neg} Eomes^{neg} MAIT cells from nine HIV-1 infected ART-untreated patients. (B) PBMCs from seven healthy controls were incubated with 10 ng/ml of IL-7 for 48 h and then stained for transcription factor expression as described in Figure 6. Significance was determined using the paired t-test.
