# Supplementary material for: Arming of MAIT Cell Cytolytic Antimicrobial Activity Is Induced by IL-7 and Defective in HIV-1 Infection
Source: PLoS Pathog. 2015 Aug 21;11(8):e1005072. doi: 10.1371/journal.ppat.1005072 (PMC4546682; doi:10.1371/journal.ppat.1005072)
Supplement: S5 Fig — (A) The frequency of MAIT cells and Vα7.2+ CD161- T cells from 20 healthy controls and 31 untreated HIV-infected patients. (B) The expression of CD38, HLA-DR, CD57, and TIM-3 was then determined on the MAIT cell population from these individuals. Significance was determined using the Mann-Whitney test. Box and whisker plots show median, IQR and the 10th to the 90th percentile. (C) Spearman’s rank correlation between CD38hi-expressing MAIT cells and the frequency of MAIT cells in the peripheral blood of 31 untreated HIV-infected patients. (PDF) [file ppat.1005072.s005.pdf]

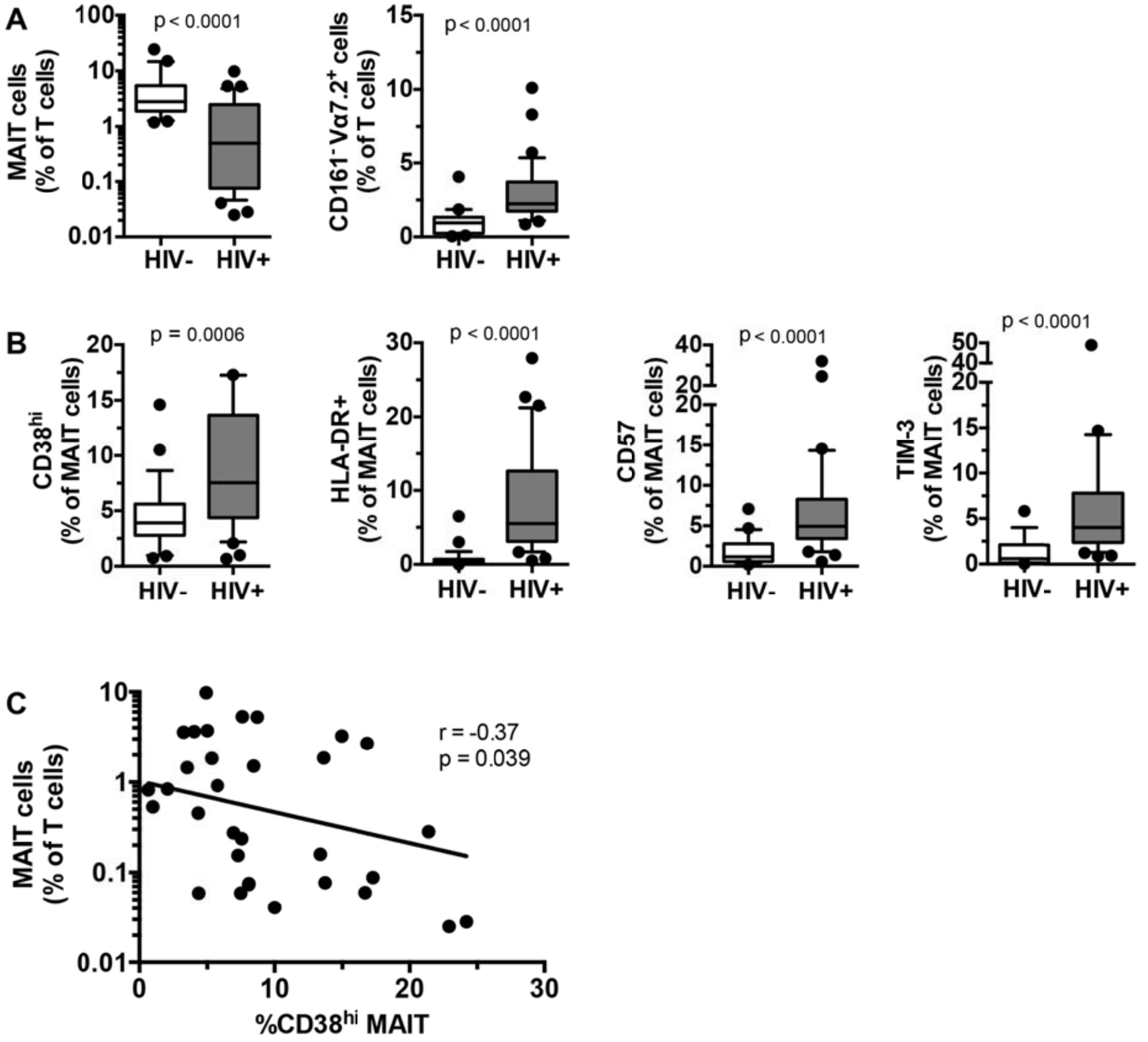

S5 Fig. MAIT cell depletion in chronically HIV-1 infected patient cohort 2 is associated with activation and exhaustion phenotypes, as previously observed for cohort 1. (A) The frequency of MAIT cells and Vα7.2^{+} CD161^{-} T cells from 20 healthy controls and 31 untreated HIV-infected patients. (B) The expression of CD38, HLA-DR, CD57, and TIM-3 was then determined on the MAIT cell population from these individuals. Significance was determined using the Mann-Whitney test. Box and whisker plots show median, IQR and the 10th to the 90th percentile. (C) Spearman's rank correlation between CD38^{hi}-expressing MAIT cells and the frequency of MAIT cells in the peripheral blood of 31 untreated HIV-infected patients.
