# Supplementary material for: Arming of MAIT Cell Cytolytic Antimicrobial Activity Is Induced by IL-7 and Defective in HIV-1 Infection
Source: PLoS Pathog. 2015 Aug 21;11(8):e1005072. doi: 10.1371/journal.ppat.1005072 (PMC4546682; doi:10.1371/journal.ppat.1005072)
Supplement: S6 Fig — Relationships between plasma IL-7 levels and CD4 counts (A), plasma viral loads (B), as well as with the MAIT cell activation markers CD38 (C), HLA-DR (D), CD57 (E), and TIM-3 (F) was assessed using Spearman’s rank correlation in 31 ART-untreated HIV-infected patients. (PDF) [file ppat.1005072.s006.pdf]

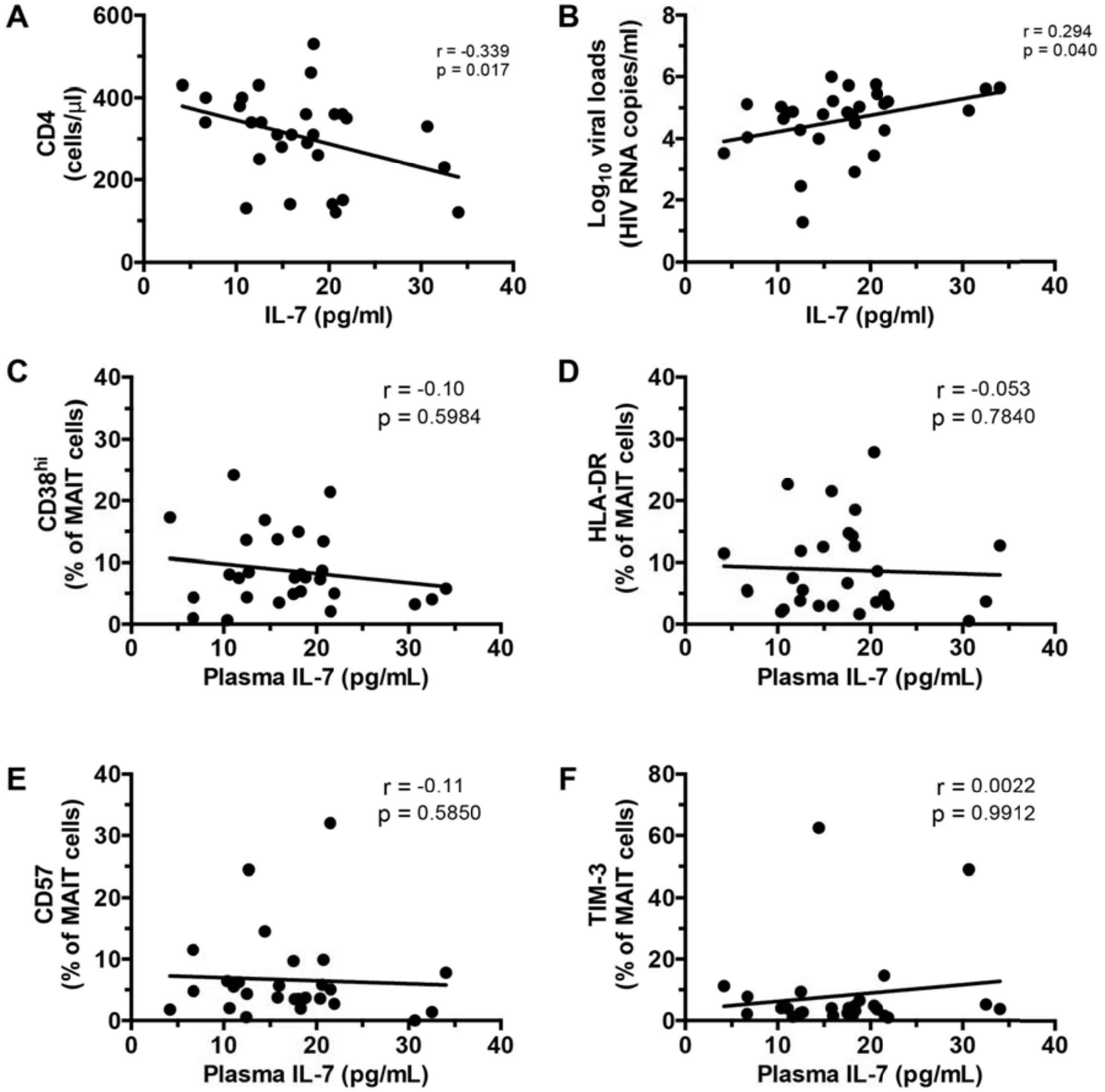

S6 Fig. Plasma IL-7 levels weakly correlate with CD4 counts and plasma viral load, but not with MAIT cell activation markers. Relationships between plasma IL-7 levels and CD4 counts (A), plasma viral loads (B), as well as with the MAIT cell activation markers CD38 (C), HLA-DR (D), CD57 (E), and TIM-3 (F) was assessed using Spearman's rank correlation in 31 ART-untreated HIV-infected patients.
